# Neurodevelopmental outcomes following prenatal cannabinoid exposure: a systematic review

**DOI:** 10.1007/s00787-026-03126-z

**Published:** 2026-07-04

**Authors:** Maria Leonor Domingues, Bruno Miguel Fonseca, Susana Isabel Sá

**Affiliations:** 1 Department of Biomedicine, Faculty of Medicine, University of Porto, Alameda Prof. Hernâni Monteiro, Porto, 4200-319 Portugal; 2 UCIBIO – Applied Molecular Biosciences Unit, Laboratório de Bioquímica, Faculdade de Farmácia, Universidade do Porto, Rua de Jorge Viterbo Ferreira 228, Porto, 4050-313 Portugal; 3 Associate Laboratory i4HB - Institute for Health and Bioeconomy, Faculdade de Farmácia, Universidade do Porto, Porto, 4050-313 Portugal; 4 RISE-Health, Faculty of Medicine, University of Porto, Alameda Prof. Hernâni Monteiro, Porto, 4200-319 Portugal

**Keywords:** Prenatal cannabis exposure, Neurodevelopmental outcomes, Executive function, Attention deficit hyperactivity disorder, Behavioural regulation, Systematic review

## Abstract

Cannabis use during pregnancy is increasing worldwide, yet evidence regarding its long-term neurodevelopmental consequences remains heterogeneous and inconclusive. This systematic review aimed to synthesise observational evidence on the association between prenatal cannabis exposure and neurodevelopmental outcomes from birth to adolescence. A systematic search of PubMed/MEDLINE, Web of Science, and PsycINFO was conducted from database inception to October 2025. Observational studies evaluating neurodevelopmental outcomes following prenatal cannabis exposure were included. Outcomes were grouped by domain and age group. Risk of bias was assessed using the Newcastle-Ottawa Scale. Due to substantial heterogeneity in exposure definitions, outcome measures, and confounder adjustment, findings were synthesised narratively. Seventy-two studies met inclusion criteria. Associations appeared to be domain-specific rather than reflecting global neurodevelopmental impairment. Behavioural dysregulation, attention-related difficulties, and executive functioning showed the most consistent associations with prenatal cannabis exposure, with 73%, 69%, and 70% of studies within these domains reporting negative associations, respectively. In contrast, most studies found no significant associations with global cognition (84%), language (92%), or motor development (60%) after adjustment for confounders. Several cohorts suggested stronger associations with heavier or persistent cannabis exposure after maternal awareness of pregnancy, indicating possible dose-response and timing effects. Current evidence does not consistently support global neurodevelopmental impairment following prenatal cannabis exposure but suggests a pattern of selective vulnerability in behavioural, attention and executive regulatory domains. Together, these findings support cautious clinical counselling and highlight the need for high-quality longitudinal studies with improved exposure and outcome measurements, and rigorous confounder control.

**Supplementary Information:**

The online version contains supplementary material available at https://doi.org/10.1007/s00787-026-03126-z.

## Introduction

Cannabis use among women of reproductive age has increased substantially in recent years, driven in part by growing social acceptance, increased accessibility, and expanding legalisation in several countries. This trend has also been observed among pregnant and breastfeeding women [1], and cannabis is now among the most commonly used psychoactive substances during pregnancy [2, 3]. Despite this increase, evidence regarding the effects of prenatal cannabinoid exposure on offspring remains limited and inconclusive. In recent years, there has also been growing interest in the potential therapeutic applications of cannabinoids, including their possible role in alleviating pregnancy-related symptoms [4]. These considerations underscore the importance of better understanding the potential effects of prenatal cannabinoid exposure on foetal development and long-term outcomes in children.

Although prenatal cannabis exposure has been strongly associated with short-term obstetric and neonatal complications, including low birth weight, stillbirth, and preterm delivery [5–7], considerably less is known about its potential long-term effects on child neurodevelopment. Some investigations have suggested associations between in utero cannabis exposure and adverse neurodevelopmental outcomes, including cognitive impairment, behavioural dysregulation, and lower intelligence scores [8, 9]. However, findings across studies remain heterogeneous and, at times, conflicting. Effect estimates vary substantially in magnitude and are often attenuated after adjustment for key confounding factors such as maternal tobacco and alcohol use, socioeconomic disadvantage, and maternal mental health conditions [10, 11]. In addition, persistent cannabis use during pregnancy may partially reflect maternal self-medication for underlying psychiatric or neurodevelopmental conditions, which themselves may independently contribute to offspring neurodevelopmental outcomes through shared genetic and environmental mechanisms.

Experimental and epidemiological evidence indicates that cannabinoids, including Δ9-tetrahydrocannabinol (THC) and cannabidiol (CBD), can cross the placental barrier and interact with the foetal endocannabinoid system, which plays a critical role in neuronal proliferation, migration, synaptogenesis, and neurotransmitter regulation during brain development [12–14]. Disruption of these tightly regulated processes during sensitive periods of gestation may plausibly influence long-term neurodevelopmental trajectories [9].

Furthermore, neuroimaging and neurobiological studies suggest that prenatal cannabinoid exposure is associated with structural and functional alterations in brain regions involved in cognition, emotional regulation, and executive functioning, including the prefrontal cortex and hippocampal formation [15–17], supporting the biological plausibility of lasting neurodevelopmental effects following prenatal cannabis exposure. Importantly, several neurodevelopmental domains potentially affected by prenatal cannabis exposure, including attentional regulation, executive functioning, impulsivity, and behavioural self-regulation, represent partially overlapping constructs supported by interconnected prefrontal and fronto-limbic neural networks. Nevertheless, these domains are commonly distinguished in developmental neuropsychology according to the predominant cognitive or behavioural process assessed and the specific instruments employed.

Despite the growing body of research in this field, important gaps remain. Existing reviews have often combined maternal, perinatal, and child outcomes, included multiple exposures, or focused on selected behavioural domains, thereby limiting their applicability to a comprehensive evaluation of offspring neurodevelopment [18]. A systematic and transparent synthesis specifically addressing neurodevelopmental outcomes following prenatal cannabis exposure is, therefore, warranted.

The aim of this systematic review is to critically evaluate and synthesise the existing literature on the association between prenatal cannabinoid exposure and neurodevelopmental outcomes in offspring, from birth to 18 years of age. Specifically, we examined evidence of observational research evaluating outcomes in multiple neurodevelopmental domains, including cognition, language, motor development, attention, executive function, behaviour, and socioemotional development, while carefully considering study design, exposure assessment, outcome measurement, confounder adjustment strategies and risk of bias. These findings aim to inform clinical counselling, public health policy, and future research on cannabis use during pregnancy.

## Methods

### Study design and protocol registration

**Fig. 1:**
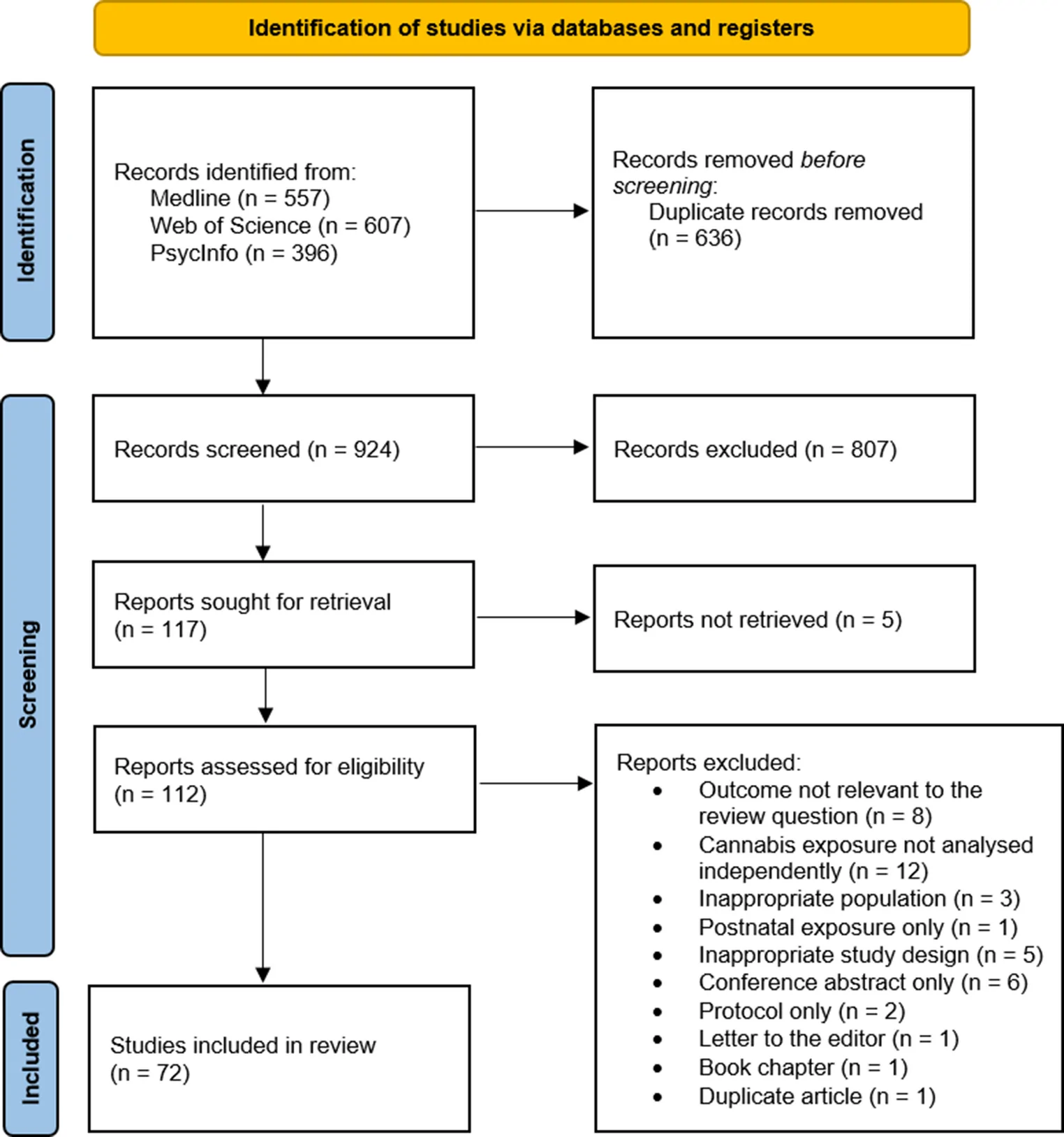
PRISMA flowchart of study selection process. (adapted from Page et al. [19])

This systematic review was conducted and reported according to the Preferred Reporting Items for Systematic Reviews and Meta-Analyses (PRISMA) 2020 guidelines (Fig. 1) [19]. The protocol was prospectively registered in PROSPERO (registration number: CRD420251184960).

### Eligibility criteria

We performed a systematic literature review according to the PICOS strategy [20]. Studies were included in this review if they satisfied the following criteria:

- Population (P): Studies including pregnant women exposed to cannabis or cannabinoid-containing products during pregnancy, and reporting neurodevelopmental outcomes in their offspring, from birth to 18 years of age.
- Intervention/Exposure (I): Prenatal exposure to cannabis or cannabinoids, including Δ9-tetrahydrocannabinol (THC), cannabidiol (CBD), or clinically diagnosed cannabis use disorder during pregnancy.
- Comparator/Control (C): Offspring with no reported prenatal cannabis exposure, or comparison groups defined by different levels of exposure (e.g., non-users, moderate users, heavy users).
- Outcomes (O): Neurodevelopmental outcomes assessed with a validated or objective measurement, at any time from birth to 18 years of age.
- Study design (S): Prospective and retrospective observational studies, published in peer-reviewed journals.

We included articles published in any language and with no restriction on publication date. Studies examining poly-substance use prenatally were included only if cannabis exposure was reported and analysed separately from other substances. Animal studies, case reports, conference abstracts, and studies not reporting neurodevelopmental outcomes were excluded.

### Information sources and search strategy

A comprehensive literature search was conducted in PubMed/MEDLINE, Web of Science and PsycINFO from database inception to October 25th, 2025. Additionally, the reference lists of review articles were hand-searched for other potentially relevant references.

The search strategy combined terms related to cannabis exposure, pregnancy and neurodevelopmental outcomes. Controlled vocabulary (MeSH terms) and free-text keywords were used. The full search strategy is provided in Supplementary Material (Table S1).

### Study selection

All identified records were imported into reference management software (Rayyan) and duplicate records were removed. Titles and abstracts were screened independently by three reviewers, and all relevant studies were identified. Full-text articles were subsequently assessed for eligibility according to predefined inclusion and exclusion criteria, and reasons for exclusion were noted (see Table S2). Discrepancies were resolved through discussion and consensus.

### Data extraction

Data were extracted independently by two reviewers using a standardised extraction form. Extracted variables included:

- Study characteristics (author(s), year of publication, country, design, patient cohort, main goal of the study).
- Sample size and population characteristics (number of exposed vs. non-exposed, children’s age, follow-up period).
- Characteristics of prenatal cannabis exposure (time/duration of cannabis exposure, exposure frequency/amount, exposure assessment method, co-exposures assessed).
- Neurodevelopmental outcomes (neurodevelopmental domain, assessment tool, age at outcome assessment).
- Findings (effect estimates, confidence intervals and confounders adjusted for).

When multiple statistical models were reported, the most fully adjusted model was extracted. Covariates commonly included maternal tobacco and alcohol use, socioeconomic variables, maternal education, maternal mental health, and other substance exposures. A detailed summary of study-specific covariate adjustment is provided in Supplementary Table S4 (Supplementary material).

### Risk of bias assessment

Risk of bias was assessed independently by two reviewers using the Newcastle–Ottawa Scale (NOS) for observational studies [21]. Studies were evaluated across three domains: selection, comparability, and outcome assessment and were categorised as low (7–9), moderate (5–6), or high (1–4) risk of bias based on NOS scores, as represented in Table S3 (Supplementary material). Disagreements were resolved through discussion.

### Data synthesis

A quantitative meta-analysis was initially planned to estimate pooled effect sizes, as stated in the registered protocol (PROSPERO CRD420251184960). However, substantial heterogeneity in exposure definitions, timing and frequency of prenatal exposure, neurodevelopmental outcome measures, and statistical adjustment strategies precluded meaningful quantitative synthesis. Additionally, variability in effect size metrics and insufficient reporting of comparable outcome data limited the feasibility of pooling results.

Therefore, findings were synthesised narratively and organised according to developmental stages and neurodevelopmental domains, including cognition, behavioural outcomes, language and communication development, attention and attention-deficit/hyperactivity disorder (ADHD)-related outcomes, motor development, executive function, and visuospatial development. Particular attention was given to study quality, risk of bias, and the degree of adjustment for key confounding variables.

## Results

### Study selection

The database search identified a total of 1560 records. After removal of duplicates, 924 records remained for title and abstract screening. Following screening, 117 reports were sought for retrieval, and 112 were assessed for full-text eligibility. Of these, 72 studies met the inclusion criteria and were included in the final synthesis.

Reasons for full-text exclusion are detailed in Table S2 (Supplementary material) and study selection process is summarised in Fig. 1.

### Characteristics of included studies and quality assessment

The 72 included studies were published between 1985 and 2025 and predominantly employed prospective or retrospective cohort designs, with a smaller number of case-control and cross-sectional studies. Sample sizes varied substantially, ranging from fewer than 100 participants to large population-based cohorts including several thousand mother-child pairs.

Some studies assessed prenatal cannabis exposure through maternal self-report during pregnancy, while others relied on biological measures (e.g., urine or meconium testing) or clinically recorded diagnoses of cannabis use disorder. The timing and frequency of exposure varied across studies, with some distinguishing between trimester-specific use, while others evaluated exposure as a binary variable (any use vs. none).

Neurodevelopmental outcomes were assessed across multiple domains, including cognition, behaviour, language and communication, attention, motor development, executive function, and visuospatial development. Outcomes were measured using standardised and validated instruments (e.g., Wechsler scales, Bayley Scales of Infant Development, Child Behaviour Checklist) or, in some cases, clinical diagnoses recorded in administrative databases.

Follow-up duration ranged from infancy to late adolescence, with several longitudinal cohorts conducting repeated assessments across developmental stages. The included studies were conducted primarily in North America and Europe, with additional contributions from Australia and other regions.

Given the heterogeneity in exposure assessment, outcome measurement, and follow-up duration, findings are synthesised narratively and presented according to neurodevelopmental domain and age group. Developmental age groups were defined according to broad neurodevelopmental stages commonly used in paediatric and developmental neuroscience research, reflecting major transitions in cognitive, behavioural, and executive maturation from infancy through adolescence [22]. This categorisation was also intended to facilitate comparison of outcomes across studies conducted at different developmental stages and to account for age-related variability in neurodevelopmental assessment and symptom expression.

Studies were therefore grouped into the following developmental periods: neonatal (0–28 days), early infancy (0–2 years), preschool (3–5 years), school age (6–12 years) and adolescence (≥ 13 years).

Tables 1, 2, 3, 4, 5, 6 and 7 present an overview of the included studies, organised by neurodevelopmental domain to facilitate comparison and interpretation. As several studies assessed more than one domain, they may be represented in more than one table.

**Table 1:** Characteristics and main findings of included studies assessing cognitive outcomes following prenatal cannabis exposure

Author	Study design	Exposure assessment methods	Comparison groups	Developmental age group	Age at assessment	Outcome assessment tools	Main findings after controlling for confounders
Fried et al. [23]	Prospective cohort	Maternal self-report	Exposure level comparison	Neonatal, early infancy and preschool age	0–4 years	McCarthy Scales and Bayley Scales of Infant Development	No significant difference was found between groups.
Richardson et al. [24]	Prospective cohort	Maternal self-report	Exposure level comparison	Early infancy	9 months and 19 months	Bayley Scales of Infant Development	Third-trimester marijuana use was significantly associated with lower Mental Development Index (MDI) scores at 9 months; however, this association was no longer evident at 19 months.
Talavera-Barber et al. [25]	Population-based prospective cohort	Maternal self-report	Any exposure vs. no exposure	Early infancy	12 months	Mullen Scales of Early Learning	No significant difference was found between groups.
Tennes et al. [26]	Prospective cohort	Maternal self-report	Exposure level comparison	Early infancy	12 months	Bayley Scales of Infant Development	No significant difference was found between groups.
Jacobson et al. [27]	Prospective cohort	Maternal self-report + Biological testing (maternal urine)	Any exposure vs. no exposure	Early infancy	12–13 months	Bayley Scales of Infant Development, Fagan Test of Infant Intelligence	No significant difference was found between groups.
Serino et al. [28]	Prospective cohort	Maternal self-report + Biological testing (maternal urine)	Any exposure vs. no exposure	Early infancy	12 months and 18–24 months of age	Bayley Scales of Infant Development	No significant difference was found between groups.
Singer et al. [29]	Prospective cohort	Maternal self-report + Biological testing (maternal urine)	Any exposure vs. no exposure	Early infancy	1.5 years	Bayley Scales of Infant Development	No significant difference was found between groups.
Fried et al. [30]	Prospective cohort	Maternal self-report	Heavy exposure vs. no exposure	Early infancy	1–2 years	Bayley Scales of Infant Development	No significant difference was found between groups.
Pleau et al. [31]	Population-based prospective cohort	Maternal self-report	Any exposure vs. no exposure	Early infancy	2 years	Bayley Scales of Infant and Toddler Development	No significant difference was found between groups.
Fried et al. [32]	Prospective cohort	Maternal self-report	Exposure level comparison	Early infancy and preschool age	4 days- 9 years	Bayley Scales of Infant Development; McCarthy Scales of Children’s Abilities	No significant difference was found between groups.
Day et al. [33]	Prospective cohort	Maternal self-report	Any exposure vs. no exposure	Preschool age	3 years	Stanford-Binet Intelligence Scale: 4th Edition (measuring verbal reasoning, abstract/visual reasoning, quantitative reasoning, and short-term memory)	Prenatal cannabis exposure was associated with lower IQ scores and significantly poorer performance in verbal reasoning, short-term memory, and abstract/visual reasoning (β ≈ −0.9 to − 8.9 points per 1 joint/day; trimester- and subgroup-dependent; *p* < 0.05).
Griffith et al. [34]	Prospective cohort	Biological testing (maternal urine) + Medical/administrative records	Any exposure vs. no exposure	Preschool age	3 years	Stanford-Binet Intelligence Scale: 4th Edition	No significant difference was found between groups.
Jarque et al. [35]	Prospective cohort	Biological testing (meconium)	Any exposure vs. no exposure	Preschool age	3 years	Bayley Scales of Infant Development	No significant difference was found between groups.
Hayes et al.[36]	Prospective cohort	Maternal self-report	Any exposure vs. no exposure	Preschool age	4–5 years	McCarthy Scales of Children’s Abilities	No significant difference was found between groups.
Moore et al. [37]	Prospective cohort	Biological testing (maternal and child urine)	Any exposure vs. no exposure	Preschool age	5 years	NIH Toolbox Cognition Battery	No significant difference was found between groups.
Smid et al. [11]	Prospective cohort	Biological testing (maternal urine)	Any exposure vs. no exposure	Preschool age	5 years	Wechsler Preschool and Primary Scale of Intelligence: 3rd Edition	No significant difference was found between groups.
Fried et al. [38]	Prospective cohort	Maternal self-report	Exposure level comparison	Preschool age	5–6 years	McCarthy Scales of Children’s Abilities; Peabody Picture Vocabulary Test-Revised	No significant difference was found between groups.
Bennett et al. [39]	Prospective cohort	Maternal self-report + Biological testing (meconium)	Any exposure vs. no exposure	Preschool and school age	4, 6, and 9 years	Stanford-Binet Intelligence Scale: 4th Edition, Short-Form	No significant difference was found between groups.
Corsi et al. [40]	Population-based retrospective cohort	Maternal self-report	Any exposure vs. no exposure	Preschool and school age	4–10 years	Clinical diagnosis (intellectual disability and learning disorders)	No significant difference was found between groups.
Goldschmidt et al. [41]	Prospective cohort	Maternal self-report	Heavy exposure vs. light/no exposure	School age	6 years	Stanford-Binet Intelligence Scale: 4th Edition	Prenatal cannabis exposure was significantly associated with reduced intellectual performance at school age (Composite IQ: β = −5.04, *p* < 0.05; Quantitative reasoning: β = −8.18, *p* < 0.01; Short-term memory: β = −4.51, *p* < 0.05).
O’Connel et al. [42]	Prospective cohort	Maternal self-report	Heavy exposure vs. no exposure	School age	6–9 years	Wechsler Intelligence Scale for Children (Revised); Wide Range Achievement Test (Revised)	No significant difference was found between groups.
Crone et al. [43]	Population-based retrospective cohort	Maternal self-report	Any exposure vs. no exposure	School age	7 years	Differential Ability Scales 2	No significant difference was found between groups.
Cioffredi et al. [44]	Population-based retrospective cohort	Maternal self-report	Any exposure vs. no exposure	School age	9–10 years	NIH Toolbox	No significant difference was found between groups.
Richardson et al. [45]	Prospective cohort	Maternal self-report	Exposure level comparison	School age	10 years	Wide Range Assessment of Memory and Learning	First-trimester heavy marijuana exposure was significantly associated with lower scores on design memory (β = −0.83, *p* < 0.05) and learning scores (β = −3.34, *p* < 0.05).
Day et al. [46]	Prospective cohort	Maternal self-report	Heavy exposure vs. light/no exposure	School age	10 years	Stanford-Binet Intelligence Scale	No significant difference was found between groups.
Goldschmidt et al. [47]	Prospective cohort	Maternal self-report	Exposure level comparison	School age	10 years	Wide Range Achievement Test- Revised (Reading, Spelling, Arithmetic), Peabody Individual Achievement Test- Revised (Reading Comprehension), and Teacher’s Evaluation	Heavy first-trimester marijuana exposure (≥ 1 joint/day) was significantly associated with poorer reading (β = −3.0, *p* < 0.05) and spelling performance (β = −3.5, *p* < 0.05) and lower teacher-rated academic functioning (β = −0.25, *p* < 0.05). No significant differences were observed among children exposed to light-to-moderate levels (< 1 joint/day) compared with non-exposed peers.
Rose-Jacobs et al. [48]	Prospective cohort	Maternal self-report + Biological testing (meconium and maternal/infant urine)	Any exposure vs. no exposure	School age	11 years	Wechsler Individual Achievement Test-Second Edition	No significant difference was found between groups.
Fried et al. [49]	Prospective cohort	Maternal self-report	Exposure level comparison	School age	9–12 years	Wechsler Intelligence Scale for Children-III	No significant difference was found between groups.
Fried et al. [50]	Prospective cohort	Maternal self-report	Exposure level comparison	School age	9–12 years	Wechsler Intelligence Scale for Children-III (Perceptual Organization Index)	Prenatal cannabis exposure was negatively associated with complex visuoperceptual tasks that require integration and analysis (*p* < 0.05).
Corsi et al. [51]	Population-based prospective cohort	Maternal self-report	Exposure level comparison	School age and adolescence	8 years (IQ), 16 years (GCSE)	Wechsler Intelligence Scale for Children for IQ and linked administrative data for GCSE scores (General Certificate of Secondary Education)	No significant difference was found between groups.
Fried et al. [52]	Prospective cohort	Maternal self-report	Heavy exposure vs. light/no exposure	Adolescence	13–16 years	WISC-III Short Form; Reading Achievement Test; Peabody Spelling Recognition; Missing Numbers; Sentence Memory	No significant difference was found between groups.
Goldschmidt et al. [53]	Prospective cohort	Maternal self-report	Heavy exposure vs. light/no exposure	Adolescence	14 years	Wechsler Individual Achievement Test	Heavy prenatal marijuana exposure (≥ 1 joint/day) during the first trimester was significantly associated with lower WIAT composite (β = −2.9, *p* < 0.05) and Basic Reading scores (β = −3.3, *p* < 0.05). No significant associations were observed for 2nd or 3rd trimester exposure.

**Table 2:** Characteristics and main findings of included studies assessing behavioural outcomes following prenatal cannabis exposure

Author	Study design	Exposure assessment methods	Comparison groups	Developmental age group	Age at assessment	Outcome assessment tools	Main findings after controlling for confounders
Tennes et al. [26]	Prospective cohort	Maternal self-report	Exposure level comparison	Neonatal	24–72 h	Brazelton Neonatal Behavioural Assessment Scale	No significant difference was found between groups.
Fried et al. [23]	Prospective cohort	Maternal self-report	Exposure level comparison	Neonatal	0–28 days	Brazelton Neonatal Assessment Scale; Prechtl Neurological Examination	Prenatal exposure was associated with increased fine tremors, exaggerated startles, and poorer habituation to visual stimuli (quantitative effect estimates were not reported).
Lester et al. [54]	Prospective cohort	Maternal self-report + Biological testing (meconium)	Exposure level comparison	Early infancy	1 month corrected age	NICU Network Neurobehavioral Scale	Prenatal cannabis exposure was associated with increased stress/withdrawal signs among children in the ‘some use’ group (*p* = 0.030) and with higher excitability among those in the ‘heavy use’ group (*p* = 0.046).
Tennes et al. [26]	Prospective cohort	Maternal self-report	Exposure level comparison	Early infancy	12 months	Bayley Infant Scale of Mental and Motor Development	No significant difference was found between groups.
Peterson et al. [55]	Prospective cohort	Maternal self-report + Biological testing + Medical/administrative records	Any exposure vs. no exposure	Early infancy	12 months	Bayley Scales of Infant Development-III; Vineland Adaptive Behaviour Scales	Prenatal cannabis exposure was significantly associated with lower general adaptive behaviour and reduced performance on the Bayley Scales of Infant and Toddler Development (*p* < 0.05).
Faden et al. [56]	Population-based prospective cohort	Maternal self-report	Any exposure vs. no exposure	Preschool age	3 years	Denver Developmental Scale and a 25-item maternal report on behaviour problems	Prenatal cannabis exposure was significantly associated with elevated fearfulness scores (*p* < 0.05).
Griffith et al. [34]	Prospective cohort	Biological testing (maternal urine) + Medical/administrative records	Any exposure vs. no exposure	Preschool age	3 years	Child Behaviour Checklist	No significant difference was found between groups.
Murnan et al. [57]	Prospective cohort	Maternal self-report + Biological testing (maternal urine)	Any exposure vs. no exposure	Preschool age	3,5 years	Child Behaviour Checklist; Bobo Interaction Task.	Prenatal cannabis exposure was significantly associated with increased sleep-related problems (β = 2.08 [− 0.08–4.24], *p* = 0.05), withdrawal symptoms (β = 2.07 [0.24–3.90], *p* = 0.02), and externalizing behaviours (β = 6.74 [0.76–12.72], *p* = 0.03), including aggressive (β = 5.73 [0.89–10.58], *p* = 0.02) and oppositional defiant behaviours (β = 2.07, *p* = 0.03).
Moore et al. [37]	Prospective cohort	Biological testing (maternal and child urine)	Any exposure vs. no exposure	Preschool age	5 years	Child Behaviour Checklist	Prenatal cannabis exposure was associated with lower internalizing behaviour scores on the CBCL, with a mean difference of − 10.2 [− 20.3 to − 0.2] points compared with non-exposed children (*p* = 0.04), suggesting fewer internalizing symptoms.
Accornero et al. [58]	Prospective cohort	Maternal self-report + Biological testing (meconium and maternal/infant urine)	Any exposure vs. no exposure	Preschool age	5 years	Achenbach Child Behaviour Checklist	No significant difference was found between groups.
DiGuiseppi et al. [59]	Retrospective cohort	Maternal self-report	Any exposure vs. no exposure	Preschool age	2–6 years	Autism Diagnostic Observation-Schedule; Autism Diagnostic Interview-Revised; Mullen Scales of Early Learning; Social Communication Questionnaire	No significant difference was found between groups.
Chatterji et al. [60]	Population-based prospective cohort	Maternal self-report	Any exposure vs. no exposure	Preschool, school age and adolescence	4–15 years	Behaviour Problems Index, based on the Achenbach Behaviour Problems Checklist.	Prenatal cannabis exposure was significantly associated with increased behaviour problems (β = 7.03; t = 8.51; *p* < 0.01).
Crone et al. [43]	Population-based retrospective cohort	Maternal self-report	Any exposure vs. no exposure	School age	7 years	Child Behaviour Checklist (CBCL); Teacher Response Form (TRF)	Prenatal cannabis exposure was associated with higher internalizing and externalizing scores on both the CBCL and TRF (*p* < 0.001).
El Marroun et al. [61]	Population-based prospective cohort	Maternal self-report + Biological testing (maternal urine)	Persistent exposure vs. no exposure	School age	7–9 years	Brief Problem Monitor; Child Behaviour Checklist; Teacher Report Form	Prenatal cannabis exposure was significantly associated with increased externalizing problems (TRF: B = 0.53 [0.29–0.77], *p* < 0.001; CBCL: B = 0.48 [0.09–0.87], *p* = 0.02), but not with internalizing problems.
Peterson et al. [55]	Population-based retrospective cohort	Medical/administrative records	Exposure level comparison	School age	8 years	ASD diagnosis based on educational classification under the IDEA (Individuals with Disabilities Education Act) database	Prenatal cannabis exposure was significantly associated with a higher prevalence of autism spectrum disorder (β = 4.37, [4.06–4.68]*p* < 0.001).
Winiger et al. [62]	Population-based prospective cohort	Maternal self-report	Exposure level comparison	School age	9–10 years	Sleep Disturbance Scale for Children, a 26-item parent report	Prenatal cannabis exposure was significantly associated with a higher prevalence of sleep disturbances, including disorders of initiating and maintaining sleep (β = 0.14, *p* < 0.01), arousal disorders (β = 0.10, *p* = 0.03), sleep–wake disorders (β = 0.16, *p* < 0.01) and excessive somnolence (β = 0.11, *p* = 0.03).
Cioffredi et al. [44]	Population-based retrospective cohort	Maternal self-report	Any exposure vs. no exposure	School age	9–10 years	Child Behaviour Checklist; Brief Problem Monitor- Teacher	Children without prenatal exposure showed significantly lower CBCL externalizing symptoms compared with the prenatal cannabis exposure group (Rate ratio = 0.72 [0.57–0.92], *p* = 0.009) and lower total problems (Rate ratio = 0.77 [0.65–0.92], *p* = 0.004).
Roffman et al. [63]	Population-based prospective cohort	Maternal self-report	Persistent exposure vs. no exposure	School age	9–10 years	Child Behaviour Checklist	Prenatal cannabis exposure was independently associated with higher child psychopathology (CBCL total problems) in both minimally and fully adjusted regression models (*p* ≤ 0.017).
Gu et al. [64]	Population-based retrospective cohort	Maternal self-report	Exposure level comparison	School age	9–10 years	Child Behaviour Checklist	Prenatal cannabis exposure after knowledge of pregnancy was significantly associated with more externalizing problems (β = 0.05, *p* < 0.05).
Gray et al. [65]	Prospective cohort	Maternal self-report	Exposure level comparison	School age	10 years	Children’s Depression Inventory	Prenatal cannabis exposure was significantly associated with increased levels of depressive symptoms (β = 2.58; t = 2.74; *p* < 0.01).
Goldschmidt et al. [66]	Prospective cohort	Maternal self-report	Exposure level comparison	School age	10 years	SNAP (Swanson, Nolan, and Pelham Rating Scale), Child Behaviour Checklist; Teacher’s Report Form	Prenatal cannabis exposure was significantly related to increased impulsivity (β = 1.0, *p* < 0.01), delinquency (OR = 2.4 [1.3–4.5], *p* < 0.01) and externalizing problems (β = 4.3, *p* < 0.01).
Vishnubhotla et al. [67]	Population-based retrospective cohort	Maternal self-report	Any exposure vs. no exposure	School age	10 years	Child Behaviour Checklist	Prenatal cannabis exposure was significantly associated with worse behavioural scores in 16 of 17 CBCL subscales, including externalizing problems (t = 4.67, *p* < 0.001), aggressive behaviour (t = 3.38, *p* < 0.001) and rule-breaking behaviour (t = 3.74, *p* < 0.001).
Corsi et al. [40]	Population-based retrospective cohort	Maternal self-report	Any exposure vs. no exposure	School age	10 years	Clinical diagnosis of autism spectrum disorder	Prenatal cannabis exposure was associated with a 50% increased risk of ASD (HR = 1.51[1.17–1.96], *p* < 0,05).
Paul et al. [68]	Population-based prospective cohort	Maternal self-report	Persistent exposure vs. no exposure	School age	9–11 years	Child Behaviour Checklist; Prodromal Questionnaire-Brief Child Version; NIH Toolbox Cognition Battery; Sleep Disturbance Scale for Children	Cannabis exposure after maternal awareness of pregnancy was significantly associated with greater psychopathology (including PLEs, attention, thought, social, and externalizing problems), even after adjustment for confounders (*p* < 0.001). In contrast, exposure occurring only before maternal awareness did not differ significantly from the unexposed group after accounting for confounding variables.
Nadler et al. [69]	Population-based prospective cohort	Maternal self-report	Any exposure vs. no exposure	School age	9–11 years	Child Behaviour Checklist	No significant differences were found between groups for either externalizing or internalizing behaviours.
Baranger et al. [70]	Population-based retrospective cohort	Maternal self-report	Persistent exposure vs. no exposure	School age and adolescence	9–14 years	Child Behaviour Checklist; Prodromal Questionnaire- Brief Child Version	Prenatal cannabis exposure was significantly associated with an increased vulnerability to broad-spectrum psychopathology (*p* < 0.02).
Tadesse et al. [71]	Population-based retrospective cohort	Medical/administrative records	Cannabis use disorder vs. no disorder	Adolescence	13–15 years	Autism spectrum disorder identified using ICD-10-AM codes F84.0–F84.9 from hospital and outpatient records	Prenatal cannabis exposure was significantly associated with an increased risk of autism spectrum disorder (ASD) (aRR = 3.56 [2.49, 5.10], *p* < 0.05).
Day et al. [46]	Prospective cohort	Maternal self-report	Heavy exposure vs. light/no exposure	Adolescence	14 years	Combined score from Child Behaviour Checklist and Self-Report Delinquency Scale	Heavy prenatal cannabis exposure was significantly associated with increased delinquent behaviour (aOR = 1.84 [1.05–2–96], *p* < 0.03).
Tadesse et al. [72]	Population-based retrospective cohort	Medical/administrative records	Cannabis use disorder vs. no disorder	Adolescence	15 years	ICD codes F91 through F91.9 (including oppositional defiant disorder and conduct disorder)	Prenatal cannabis exposure was significantly associated with an increased risk of disruptive behavioural disorders (RR = 3.56 [2.45–5.05], *p* < 0.001).
Nutor et al. [73]	Population-based prospective cohort	Maternal self-report + Biological testing + Medical/administrative records	Any exposure vs. no exposure	Early infancy, preschool, school age and adolescence	1–18 years	ASD Diagnosis (parent report, ICD codes, or ADOS-2); Child Behaviour Checklist; Social Responsiveness Scale	No significant difference was found between groups.

**Table 3:** Characteristics and main findings of included studies assessing language and communication outcomes following prenatal cannabis exposure

Author	Study design	Exposure assessment methods	Comparison groups	Developmental age group	Age at assessment	Outcome assessment tools	Main findings after controlling for confounders
Salzwedel et al. [74]	Prospective cohort	Maternal self-report + Biological testing + Medical/administrative records	Any exposure vs. no exposure	Early infancy	3 months	Bayley Scales of Infant and Toddler Development III	No significant difference was found between groups.
Watts et al. [75]	Prospective cohort	Maternal self-report	Any exposure vs. no exposure	Early infancy	12 months	Ages and Stages Questionnaire- Third Edition	No significant difference was found between groups.
Serino et al. [28]	Prospective cohort	Maternal self-report + Biological testing (maternal urine)	Any exposure vs. no exposure	Early infancy	12 months and 18–24 months of age	Bayley Scales of Infant Development- 3rd Edition	No significant difference was found between groups.
Fried et al. [30]	Prospective cohort	Maternal self-report	Heavy exposure vs. no exposure	Early infancy	1–2 years	Bayley Scales of Infant Development; Reynell Developmental Language Scales	No significant difference was found between groups.
Pleau et al. [31]	Population-based prospective cohort	Maternal self-report	Any exposure vs. no exposure	Early infancy	2 years	MacArthur-Bates Communicative Development Inventories	No significant difference was found between groups.
Jarque et al. [35]	Prospective cohort	Biological testing (meconium)	Any exposure vs. no exposure	Preschool age	3 years	Bayley Scales of Infant and Toddler Development- 3rd Edition	No significant difference was found between groups.
Fried et al. [32]	Prospective cohort	Maternal self-report	Exposure level comparison	Preschool age	4 years	Reynell Developmental Language Scales	No significant difference was found between groups.
Fried et al. [76]	Prospective cohort	Maternal self-report	Exposure level comparison	Preschool age	4 years	Reynell Developmental Language Scales; Peabody Picture Vocabulary Test	Prenatal cannabis exposure was significantly associated with lower verbal and memory scores (*p* < 0.05). Effect sizes not reported.
Avalos et al. [77]	Population-based retrospective cohort	Maternal self-report + Biological testing (maternal urine)	Exposure level comparison	Preschool age	5 years	ICD-10 diagnosis codes and CPT codes ascertained from Electronic Health Records (referring to speech and language disorders)	No significant difference was found between groups.
Fried et al. [38]	Prospective cohort	Maternal self-report	Exposure level comparison	Preschool age	5–6 years	Peabody Picture Vocabulary Test-Revised	No significant difference was found between groups.
O’Connel et al. [42]	Prospective cohort	Maternal self-report	Heavy exposure vs. no exposure	School age	6–9 years	Test of Language Development- Primary	No significant difference was found between groups.
Fried et al. [78]	Prospective cohort	Maternal self-report	Exposure level comparison	School age	9–12 years	Peabody Picture Vocabulary Test; Fluency Test; Woodcock Reading Mastery Test; Oral Cloze Task; Pseudoword Task	No significant difference was found between groups.

**Table 4:** Characteristics and main findings of included studies assessing attention and ADHD-related outcomes following prenatal cannabis exposure

Author	Study design	Exposure assessment methods	Comparison groups	Developmental age group	Age at assessment	Outcome assessment tools	Main findings after controlling for confounders
Fried et al. [32]	Prospective cohort	Maternal self-report	Exposure level comparison	Early infancy, preschool and school age	4 days- 9 years	Computerized Vigilance Tasks; Conners’ Parent Rating Scale	Prenatal cannabis exposure was associated with sustained attention deficits and increased distractibility, emerging mainly from preschool/school age (≈ 4–6 years) onward. Effect sizes not reported.
Noland et al. [79]	Prospective cohort	Maternal self-report + Biological testing (meconium and maternal/infant urine)	Any exposure vs. no exposure	Preschool age	4 years	Continuous Performance Task; Picture Deletion Task	Prenatal cannabis exposure was significantly associated with impaired sustained attention on the Picture Deletion Task (PDT), reflected by increased omission errors (β = 0.29, t = 1.9, *p* = 0.06)
Smid et al. [11]	Prospective cohort	Biological testing (maternal urine)	Any exposure vs. no exposure	Preschool age	4 years	Conners’ Rating Scales- Revised	Prenatal cannabis exposure was significantly associated with worse attention scores (Adjusted difference in medians = 6.0 [1.11–10.89], *p* < 0.05).
Fried et al. [80]	Prospective cohort	Maternal self-report	Exposure level comparison	Preschool age	5 years	Gordon Diagnostic System (computerized vigilance task); Conners’ Parent Rating Scale- 48	Prenatal cannabis exposure was significantly associated with increased omission errors on the vigilance task, suggesting a deficit in sustained attention (*p* < 0.05). Exposed children were also rated higher by their parents on the impulsivity/hyperactivity scale (*p* < 0.05).
Corsi et al. [40]	Population-based retrospective cohort	Maternal self-report	Any exposure vs. no exposure	Preschool and school age	4–10 years	Clinical diagnosis (ADHD)	No significant difference was found between groups.
Young-Wolf et al. [81]	Population-based retrospective cohort	Maternal self-report + Biological testing (maternal urine)	Exposure level comparison	Preschool and school age	4–11 years	Electronic Health Record data based on ICD-10-CM diagnostic codes and/or ADHD medication fills	No significant difference was found between groups.
Corsi et al. [51]	Population-based prospective cohort	Maternal self-report	Exposure level comparison	School age	7 years	Strengths and Difficulties Questionnaire for hyperactivity	No significant difference was found between groups.
Cioffredi et al. [44]	Population-based retrospective cohort	Maternal self-report	Any exposure vs. no exposure	School age	9–10 years	Child Behaviour Checklist; Brief Problem Monitor- Teacher	Prenatal cannabis exposure was significantly associated with increased attention problems, based on both parent- and teacher-report (*p* < 0.01).
Goldschmidt et al. [66]	Prospective cohort	Maternal self-report	Exposure level comparison	School age	10 years	Swanson, Nolan, and Pelham Rating Scale; Child Behaviour Checklist; Teacher’s Report Form	Prenatal cannabis exposure was significantly related to increased hyperactivity (β = 1.2, *p* < 0.001), and inattention symptoms (β = 0.9, *p* < 0.01).
Vishnubhotla et al. [67]	Population-based retrospective cohort	Maternal self-report	Any exposure vs. no exposure	School age	10 years	Child Behaviour Checklist	Prenatal cannabis exposure was significantly associated with attention related problems (t = 4.28, *p* < 0.001) and ADHD (t = 4.67, *p* < 0.001).
Ryan et al. [82]	Population-based prospective cohort	Maternal self-report	Any exposure vs. no exposure	School age	9–13 years	Child Behaviour Checklist DSM-5-oriented scales	Prenatal cannabis exposure was significantly associated with a twofold increase in the odds of ADHD symptoms compared with no exposure (aOR = 2.097 [1.152–3.817], *p* < 0.05).
Fried et al. [83]	Prospective cohort	Maternal self-report	Exposure level comparison	Adolescence	13–16 years	Continuous Performance Test; Stroop Test; Knox Cube Test	Prenatal cannabis exposure was significantly associated with reduced stability and consistency of attention over time (*p* < 0.01).
Tchuente et al. [84]	Population-based retrospective cohort	Maternal self-report	Exposure level comparison	Adolescence	18 years	ADHD diagnosis (ICD-9: 314.0 or ICD-10: F90.9) in medical databases; prescriptions for ADHD medication	No significant difference was found between groups.

**Table 5:** Characteristics and main findings of included studies assessing motor development following prenatal cannabis exposure

Author	Study design	Exposure assessment methods	Comparison groups	Developmental age group	Age at assessment	Outcome assessment tools	Main findings after controlling for confounders
Peterson et al. [55]	Prospective cohort	Maternal self-report + Biological testing + Medical/administrative records	Any exposure vs. no exposure	Early infancy	12 months	Bayley Scales of Infant Development–III	Bayley-III gross motor scores were lower in exposed infants compared with controls (Any exposure: 8.95 ± 3.17 vs. controls: 11.59 ± 2.32, *p* = 0.001).
Tennes et al. [26]	Prospective cohort	Maternal self-report	Exposure level comparison	Early infancy	12 months	Bayley Scales of Infant Development	No significant difference was found between groups.
Serino et al. [28]	Prospective cohort	Maternal self-report + Biological testing (maternal urine)	Any exposure vs. no exposure	Early infancy	12 months and 18–24 months of age	Bayley Scales of Infant Development – III	No significant difference was found between groups.
Fried et al. [23]	Prospective cohort	Maternal self-report	Exposure level comparison	Early infancy	1–2 years	Bayley Scales of Infant Development, Prechtl Neurological Examination	No significant difference was found between groups.
Richardson et al. [24]	Prospective cohort	Maternal self-report	Exposure level comparison	Early infancy	9–19 months	Bayley Scales of Infant Development	No significant difference was found between groups.
Pleau et al. [31]	Population-based prospective cohort	Maternal self-report	Any exposure vs. no exposure	Early infancy	2 years	Bayley Scales of Infant Development	No significant difference was found between groups.
Chandler et al. [85]	Prospective cohort	Maternal self-report	Exposure level comparison	Preschool age	3 years	An adapted motor assessment based on the Gross Motor Scale and the Bruininks-Oseretsky Test of Motor Proficiency	No significant difference was found between groups.
Jarque et al. [35]	Prospective cohort	Biological testing (meconium)	Any exposure vs. no exposure	Preschool age	3 years	Bayley Scales of Infant Development	Prenatal cannabis exposure was associated with poorer motor development scores. Exposed infants had lower Bayley motor composite scores and lower total motor scale scores. Significant reductions were also observed in fine and gross motor skills (*p* = 0.012).
Fried et al. [76]	Prospective cohort	Maternal self-report	Exposure level comparison	Preschool age	3 years	Pegboard Test	Prenatal cannabis exposure was significantly associated with reduced composite motor scores (F(2,125) = 4.1, *p* < 0.05).
Faden et al. [56]	Population-based prospective cohort	Maternal self-report	Any exposure vs. no exposure	Preschool age	3 years	Denver Developmental Scale	Prenatal cannabis exposure was significantly associated with reduced gross motor scores (*p* < 0.05).

**Table 6:** Characteristics and main findings of included studies assessing executive functioning following prenatal cannabis exposure

Author	Study design	Exposure assessment methods	Comparison groups	Developmental age group	Age at assessment	Outcome assessment tools	Main findings after controlling for confounders
Murnan et al. [57]	Prospective cohort	Maternal self-report + Biological testing (maternal urine)	Any exposure vs. no exposure	Preschool age	3,5 years	NIH Toolbox; Behaviour Rating Inventory of Executive Function- Preschool; Toy Behind Barrier Task	No significant differences were found between groups.
Noland et al. [86]	Prospective cohort	Maternal self-report + Biological testing (meconium)	Heavy exposure vs. light/no exposure	Preschool age	4 years	Tapping Inhibition task; Category Fluency (McCarthy Scales adaptation); Motor-Planning task (Welsh et al. adaptation)	No significant difference was found between groups on any of the executive functioning tasks (tapping inhibition, fluency, or motor planning).
Leech et al. [87]	Prospective cohort	Maternal self-report	Exposure level comparison	School age	6 years	Continuous Performance Task- 3	Second-trimester cannabis exposure was significantly associated with a higher number of commission errors, indicative of increased impulsivity (β = 0.13, *p* < 0.01).
Fried et al. [32]	Prospective cohort	Maternal self-report	Exposure level comparison	School age	6–9 years	Computerized Vigilance Tasks; Conners’ Parent Rating Scale	Exposed children demonstrated significantly higher distractibility and greater difficulties with planning, organised search, and impulse control (*p* < 0.05).
Richardson et al. [45]	Prospective cohort	Maternal self-report	Exposure level comparison	School age	10 years	Continuous Performance Test-Letters (CPT-II)	Second-trimester prenatal cannabis exposure was associated with increased impulsivity, predicting more CPT-II commission errors specifically in Block 3 (fatigue phase), (b = 1.86, *p* < 0.05).
Fried et al. [49]	Prospective cohort	Maternal self-report	Exposure level comparison	School age	9–12 years	Gordon Diagnostic Delay and Vigilance Tasks; Category Test; Auditory Working Memory Test; Fluency Test	Prenatal cannabis exposure was significantly associated with poorer performance on executive function tasks involving impulse control and visual analysis/hypothesis testing (*p* < 0.01).
Fried et al. [50]	Prospective cohort	Maternal self-report	Exposure level comparison	School age	9–12 years	Wechsler Intelligence Scale for Children-III (Perceptual Organization Index)	Prenatal cannabis exposure was negatively associated with complex visuoperceptual tasks that require integration and analysis (t = − 2.2, *p* < 0.05).
Rose-Jacobs et al. [88]	Prospective cohort	Maternal self-report + Biological testing (meconium and maternal/infant urine)	Exposure level comparison	School age and adolescence	12–14 years	Delis-Kaplan Executive Function System	Prenatal cannabis exposure was associated with poorer executive functioning, reflected by significantly lower performance on the Design Fluency Switching condition (*p* < 0.05).
Fried et al. [52]	Prospective cohort	Maternal self-report	Heavy exposure vs. light/no exposure	Adolescence	13–16 years	Wisconsin Card Sorting Test; Peabody Spelling Recognition Test; Stroop Test; Abstract Designs Test	Prenatal cannabis exposure was associated with poorer performance on tasks requiring visual memory, analysis, and integration. Exposed adolescents showed longer response latency on the Abstract Designs task (53.7 ± 1.8 vs. 49.2 ± 0.8, *p* < 0.05) and lower scores on the Peabody Spelling recognition task (97.3 ± 2.2 vs. 102.3 ± 1.0; *p* < 0.05) (*p* < 0.05).
Rose-Jacobs et al. [89]	Prospective cohort	Maternal self-report + Biological testing (meconium and maternal/infant urine)	Exposure level comparison	Adolescence	16–18 years	Behaviour Rating Inventory of Executive Functioning-Teacher Form	Heavier prenatal cannabis exposure was unexpectedly associated with better executive functioning, reducing odds of clinically significant executive dysfunction (OR = 0.3; *p* = 0.04).

**Table 7:** Characteristics and main findings of included studies assessing visuospatial development following prenatal cannabis exposure

Author	Study design	Exposure assessment methods	Comparison groups	Developmental age group	Age at assessment	Outcome assessment tools	Main findings after controlling for confounders
Bailey et al. [90]	Prospective cohort	Maternal self-report + Biological testing (maternal urine and neonatal urine, meconium or cord blood testing)	Persistent exposure vs. 1 st trimester-exposure-only/no exposure	Early infancy	15 months	Infant Toddler Sensory Profile	Prenatal cannabis exposure was associated with a twofold increased likelihood of high visual stimulation seeking (aOR = 1.94 [1.01–3.72]).
Griffith et al. [34]	Prospective cohort	Biological testing (maternal urine) + Medical/administrative records	Any exposure vs. no exposure	Preschool age	3 years	Stanford-Binet Intelligence Scale: Fourth Edition (SBIS)	Prenatal cannabis exposure was a significant predictor of poorer abstract/visual reasoning, explaining 13% of variance (B = − 0.68, *p* < 0.001).
Fried et al. [23]	Prospective cohort	Maternal self-report	Exposure level comparison	Preschool age	4 years	Visual Evoked Potential	Prenatal cannabis exposure was significantly associated with longer latencies in visual evoked potentials and significantly greater response variability, suggesting delayed visual system maturation.
Chakraborty et al. [91]	Prospective cohort	Maternal self-report + Biological testing (meconium)	Exposure level comparison	Preschool age	4.5 years	Random dot kinematograms using a 2-down-1-up adaptive staircase test to measure motion coherence thresholds	Prenatal cannabis exposure was significantly associated with better global motion perception, reflected by lower motion coherence thresholds compared with non-exposed controls (46 ± 20% vs. 63 ± 25%, t(163) = 3.52, *p* = 0.001).
O’Connel et al. [42]	Prospective cohort	Maternal self-report	Heavy exposure vs. no exposure	School age	6–9 years	Test of Visual Perceptual Skills; Beery-Buktenica Developmental Test of Visual-Motor Integration	No significant difference was found between groups.
Hiraoka et al. [92]	Population-based prospective cohort	Maternal self-report	Any exposure vs. no exposure	School age	9–13 years	Little Man Test for visuo-spatial ability	Prenatal cannabis exposure was associated with attenuated development in visuo-perceptual processing, reflected by smaller increases over time. Simple slope analyses showed greater improvement in non-exposed children (b = 0.154) compared with cannabis-exposed children (b = 0.135), (*p* < 0.01).

Table 8 summarises the distribution and direction of associations between prenatal cannabis exposure and neurodevelopmental outcomes across domains and developmental stages.

**Table 8:** Distribution and direction of associations between prenatal cannabis exposure and neurodevelopmental outcomes across developmental domains and age groups

Developmental domain	Age group	Number of studies finding no association	Number of studies finding a negative association	Number of studies finding a positive association	Total
Cognition	Neonatal	1 (100%)	0	0	1
	Early infancy	10 (100%)	0	0	10
	Preschool age	10 (91%)	1 (9%)	0	11
	School age	10 (77%)	3 (23%)	0	13
	Adolescence	2 (67%)	1 (33%)	0	3
	All age groups	27 (84%)	5 (16%)	0	32
Behaviour	Neonatal	1 (50%)	1 (50%)	0	2
	Early infancy	1 (33%)	2 (67%)	0	3
	Preschool age	3 (43%)	3 (43%)	1 (14%)	7
	School age	1 (7%)	14 (93%)	0	15
	Adolescence	1 (20%)	4 (80%)	0	5
	All age groups	7 (23%)	22 (73%)	1 (3%)	30
Language and communication	Early infancy	5 (100%)	0	0	5
	Preschool age	4 (80%)	1 (20%)	0	5
	School age	2 (100%)	0	0	2
	All age groups	11 (92%)	1 (8%)	0	12
Attention/ADHD	Preschool age	3 (50%)	3 (50%)	0	6
	School age	2 (29%)	5 (71%)	0	7
	Adolescence	1 (50%)	1 (50%)	0	2
	All age groups	4 (31%)	9 (69%)	0	13
Motor development	Early infancy	5 (83%)	1 (17%)	0	6
	Preschool age	1 (25%)	3 (75%)	0	4
	All age groups	6 (60%)	4 (40%)	0	10
Executive function	Preschool age	2 (100%)	0	0	2
	School age	0	6 (100%)	0	6
	Adolescence	0	2 (67%)	1 (33%)	3
	All age groups	2 (20%)	7 (70%)	1 (10%)	10
Visuospatial development	Early infancy	0	1 (100%)	0	1
	Preschool age	0	2 (66%)	1 (33%)	3
	School age	1 (50%)	1 (50%)	0	2
	All age groups	1 (17%)	4 (67%)	1 (17%)	6

This table summarises the direction of associations reported in the included studies according to neurodevelopmental domain and age at assessment. For each domain, studies were categorised as reporting no association, a negative association (indicating worse outcomes among exposed offspring), or a positive association (indicating better outcomes among exposed offspring) after adjustment for confounding factors. Studies assessing multiple developmental stages or multiple domains are represented in each relevant category. Percentages refer to the proportion of studies within each domain and age group reporting a given direction of association

Developmental age groups were defined as follows: neonatal (0–28 days), early infancy (0–2 years), preschool age (3–5 years), school age (6–12 years), and adolescence (≥13 years)

Risk of bias assessments of included studies are reported in Table S3. Of the 72 included studies, 70 scored between 7 and 9 on the NOS, indicating overall high methodological quality. Only 2 studies were classified as moderate quality.

## Neurodevelopmental outcomes by domain

### Cognitive outcomes

Of the 72 included studies, 32 assessed cognitive outcomes following prenatal cannabis exposure, making cognition the most frequently investigated neurodevelopmental domain (Table 1). Cognitive performance was assessed using standardised developmental and intelligence measures, including the Bayley Scales of Infant Development, Stanford-Binet and Wechsler Intelligence Scales, as well as academic achievement and memory assessments.

Findings across studies were heterogeneous. As summarised in Tables 8 and 84% of studies (27/32) did not identify a significant association between prenatal cannabis exposure and global cognitive outcomes after adjustment for relevant confounding factors, whereas 16% (5/32) reported negative associations. No studies reported positive associations. The pattern of associations varied across developmental stages: no significant associations were reported in neonatal or early infancy studies, whereas negative findings were observed in approximately 10% of preschool studies, 23% of school-age studies and one third of studies assessing adolescents (Table 8).

Reported effects generally involved modest reductions in specific domains, including IQ, verbal reasoning, memory, and academic achievement, particularly among children exposed to heavier levels of cannabis use during early pregnancy [45, 47, 53]. However, observed effect sizes were generally small and frequently attenuated after adjustment for key confounding factors, including maternal tobacco and alcohol use, socioeconomic disadvantage, and maternal mental health conditions. Importantly, no clear or consistent differences were identified in the scope of confounder adjustment between studies reporting significant associations and those reporting null findings. While many studies adjusted for similar core confounders, including maternal substance use, socioeconomic status, and maternal mental health, the extent and operationalisation of these variables varied considerably across studies, limiting direct comparability.

Overall, although isolated studies suggest subtle differences in specific cognitive domains, the available evidence does not consistently support a clear and robust independent association between prenatal cannabis exposure and clinically meaningful long-term cognitive impairment.

### Behavioural outcomes

Of the 72 included studies, 30 assessed behavioural outcomes following prenatal cannabis exposure, making behaviour one of the most frequently examined neurodevelopmental domains (Table 2). Behavioural functioning was primarily evaluated using standardised instruments, including the Child Behaviour Checklist (CBCL), Teacher Report Form (TRF), Brief Problem Monitor (BPM), and related validated scales, as well as clinical diagnoses recorded in administrative databases, such as disruptive behavioural disorders and autism spectrum disorder (ASD).

A more consistent pattern of association emerged within this domain. As summarised in Tables 8 and 73% of studies (22/30) reported a negative association between prenatal cannabis exposure and behavioural outcomes, whereas 23% (7/30) found no association and 3% (1/30) reported a positive association. Negative associations were more frequently reported at older ages, increasing from around half of neonatal studies to two thirds of early infancy and preschool studies and to 93% of school-age and 80% of adolescent studies (Table 8).

Across studies, a consistent pattern emerged linking prenatal cannabis exposure with increased behavioural dysregulation, particularly externalising problems. Multiple longitudinal cohorts reported higher levels of aggression, impulsivity, delinquent behaviour and oppositional symptoms among exposed children and adolescents. Some investigations also identified an increased risk of clinically diagnosed disruptive behavioural disorders, autism spectrum disorder or sleep disturbances. In several cohorts, heavier exposure and exposure that persisted after maternal awareness of pregnancy were associated with stronger effect estimates, suggesting a potential dose-response relationship [46, 64, 68, 89]. This may also reflect residual familial or psychiatric confounding, including maternal self-medication for underlying conditions such as ADHD, anxiety, or other psychiatric disorders, which themselves are independently associated with increased neurodevelopmental risk in offspring through shared genetic and environmental mechanisms.

Although some well-controlled studies reported null findings after comprehensive adjustment, and isolated investigations described reduced internalising symptoms or improved behavioural regulation, the overall body of evidence suggests that prenatal cannabis exposure may be associated with an increased risk of behavioural problems across childhood and adolescence. Nevertheless, the independence and clinical magnitude of these effects remain influenced by methodological limitations and potential residual confounding, including maternal psychiatric conditions and familial and genetic vulnerability.

### Language and communication

Of the 72 included studies, 12 assessed language and communication outcomes following prenatal cannabis exposure (Table 3). Language development was evaluated using standardised instruments, including the Bayley Scales of Infant and Toddler Development, Reynell Developmental Language Scales, Peabody Picture Vocabulary Test (PPVT), MacArthur–Bates Communicative Development Inventories, and other validated language assessments. Some studies also relied on diagnostic codes extracted from electronic health records.

Across studies, findings were largely consistent in showing no significant association between prenatal cannabis exposure and language or communication outcomes after adjustment for relevant confounders. As summarised in Tables 8 and 92% of studies (11/12) reported no significant association between prenatal cannabis exposure and language or communication outcomes after adjustment for confounding factors, whereas 8% (1/12) identified a negative association. No studies reported positive associations. Most investigations were conducted during early infancy and preschool period and did not identify differences in receptive or expressive language performance between exposed and non-exposed children. Similarly, studies extending into school age reported no significant associations with language development or reading-related skills. Only one study reported significantly lower verbal scores among exposed children [76]; however, this finding was not consistently replicated across other cohorts. Although adjustment included broadly similar key confounders, variation in adjustment strategies and the possibility of residual confounding remain.

Overall, the available evidence does not support a clear or consistent association between prenatal cannabis exposure and language or communication impairments.

### Attention-deficit/hyperactivity disorder (ADHD) or attention-related outcomes

Of the 72 included studies, 13 specifically assessed attention-related outcomes or ADHD following prenatal cannabis exposure (Table 4). Attention and hyperactivity were evaluated using standardised behavioural rating scales, including the Conners’ Rating Scales, Child Behaviour Checklist, Strengths and Difficulties Questionnaire, and computerised attention tasks such as the Continuous Performance Test, as well as clinical diagnoses recorded in electronic health databases.

As summarised in Tables 8 and 69% of studies (9/13) reported a negative association between prenatal cannabis exposure and attention-related outcomes, whereas 31% (4/13) found no association. No studies reported positive associations. Negative associations were observed across developmental stages, with approximately half of preschool and adolescent studies and two thirds of school-age studies reporting adverse attention-related outcomes (Table 8). Across cohorts, findings most consistently involved deficits in sustained attention, accompanied by higher levels of hyperactivity symptoms. Several investigations reported increased omission errors on vigilance tasks, reduced attentional stability over time, and elevated parent- or teacher-reported attention problems among exposed children. However, associations were less consistent when ADHD was defined using formal clinical diagnoses rather than behavioural or neuropsychological measures [40, 81, 84].

Overall, the available evidence suggests that prenatal cannabis exposure may be associated with attention deficits and ADHD-related symptoms, particularly when assessed using behavioural or neuropsychological measures. Nevertheless, variability across study designs and outcome definitions limits firm conclusions regarding clinically diagnosed ADHD.

### Motor development outcomes

Of the 72 included studies, 10 assessed motor development following prenatal cannabis exposure (Table 5). Motor outcomes were primarily evaluated in early infancy and preschool years using standardised developmental instruments, most commonly the Bayley Scales of Infant Development (BSID), as well as the Prechtl Neurological Examination, Denver Developmental Screening Test, Pegboard Test, and adapted gross motor proficiency scales.

As summarised in Tables 8 and 60% of studies (6/10) reported no significant association between prenatal cannabis exposure and motor outcomes after adjustment for confounders, whereas 40% (4/10) identified negative associations. No studies reported positive associations. Findings across investigations were mixed. Most early infancy studies did not detect differences after adjustment, whereas adverse findings were more frequently reported in preschool-aged children (Table 8). Where negative associations were observed, they more commonly involved reduced gross or composite motor scores, with gross motor skills appearing more frequently affected than fine motor abilities [55, 56].

Overall, the available evidence does not demonstrate a consistent or robust association between prenatal cannabis exposure and early motor development. Although some findings suggest possible subtle impairments, particularly in gross motor functioning, the heterogeneity of results and methodological variability limit firm conclusions. These include variability in exposure assessment methods, timing and intensity of cannabis exposure and heterogeneity in neurodevelopmental assessment tools.

### Executive function

Of the 72 included studies, 10 specifically evaluated executive functioning following prenatal cannabis exposure (Table 6). Executive abilities were assessed using standardised neuropsychological tasks, including the Continuous Performance Test (CPT), Delis–Kaplan Executive Function System (D-KEFS), Wisconsin Card Sorting Test (WCST), Stroop Test, and other measures of planning, inhibition, organised search, working memory, and impulse control.

As summarised in Tables 8 and 70% of studies (7/10) reported a negative association between prenatal cannabis exposure and executive functioning, whereas 20% (2/10) found no association and 10% (1/10) found a positive association. Findings varied across developmental stages: studies conducted during the preschool period did not identify significant differences after adjustment for confounders, whereas studies in school-age children and adolescents consistently reported negative associations. Several longitudinal cohorts reported increased impulsivity, higher commission errors on vigilance tasks, greater distractibility, and poorer performance on tasks requiring planning, hypothesis testing, cognitive flexibility, and visual integration among exposed children. Notably, these deficits were often observed in specific executive domains even when general intelligence measures were not significantly affected, suggesting that prenatal cannabis exposure may be associated with alterations in higher-order regulatory processes, potentially involving prefrontal regulatory networks, rather than global cognitive impairment.

Overall, the distribution of findings indicates a relatively consistent association between prenatal cannabis exposure and executive dysfunction, particularly in older children and adolescents and in domains related to impulse control, planning and cognitive flexibility. However, the limited number of studies and the possibility of residual confounding warrant cautious interpretation.

### Visuospatial development

Of the 72 included studies, 6 evaluated visuospatial development following prenatal cannabis exposure (Table 7). Visuospatial abilities were assessed using heterogeneous measures, including visual evoked potentials (VEP), motion coherence tasks, the Test of Visual Perceptual Skills (TVPS), the Beery–Buktenica Developmental Test of Visual-Motor Integration (VMI), the Little Man Test (LMT), and sensory processing questionnaires in early infancy.

Findings were heterogeneous and domain-specific. As summarised in Tables 8 and 67% of studies (4/6) reported a negative association between prenatal cannabis exposure and visuospatial outcomes, whereas 17% (1/6) found no association and 17% (1/6) reported positive findings. Given the small number of studies and variability in outcome definitions and assessment methods, conclusions remain limited. Where adverse findings were reported, results suggested potential alterations in sensory or visual processing trajectories rather than consistent impairments in visuospatial performance.

Overall, the available evidence suggests possible visuoperceptual differences following prenatal cannabis exposure; however, the small number of investigations and the variability in assessment methods limit firm conclusions.

## Discussion

### Summary of evidence

This systematic review synthesises evidence from 72 observational studies examining the association between prenatal cannabis exposure and neurodevelopmental outcomes from birth through adolescence. Taken together, results suggest a domain-specific pattern of vulnerability rather than global neurodevelopmental impairment. Behavioural dysregulation, attention-related difficulties, and executive dysfunction showed the most consistent associations with prenatal cannabis exposure, whereas global cognitive performance, language development, and early motor outcomes were more frequently reported as unaffected after adjustment for confounding factors.

It is also important to acknowledge the considerable conceptual overlap between attentional regulation, executive functioning, and behavioural self-regulation, particularly given the shared involvement of higher-order prefrontal regulatory systems. Studies were categorized according to the primary construct explicitly assessed by the original authors and the main assessment tools used, while recognizing that several neuropsychological tasks may capture overlapping executive and attentional processes.

The distribution of findings across domains suggests that higher-order regulatory processes may be particularly susceptible to prenatal cannabis exposure. Executive functioning, impulse control, and behavioural regulation depend on the prolonged maturation of fronto-striatal and fronto-limbic circuits, especially those involving the prefrontal cortex, which continues to develop throughout childhood and adolescence [93]. Disruption of these networks during critical developmental windows may therefore contribute to the behavioural and attentional differences observed across several cohorts. Although direct causal inference cannot be established from observational evidence alone, the convergence of findings across multiple cohorts strengthens the biological plausibility of selective vulnerability within prefrontal regulatory systems.

Importantly, several studies suggested that associations may depend on the intensity and timing of exposure. Adverse findings were more frequently observed in cohorts reporting heavier or persistent cannabis use, particularly daily or near-daily consumption [47, 53, 89]. In some studies, exposure continuing after maternal awareness of pregnancy was associated with stronger behavioural and attention-related outcomes compared with exposure limited to early gestation [24, 45, 64, 68, 87]. Trimester-specific analyses further indicated that first- and second-trimester exposure may be particularly relevant for executive and cognitive domains in certain cohorts [45, 53, 87]. Although not uniformly replicated, these patterns raise the possibility of dose-response relationships and developmental timing effects, suggesting that neurodevelopmental consequences may depend on both exposure level and gestational window.

It is important to emphasise that only three included studies reported positive or apparently beneficial associations following prenatal cannabis exposure, making these findings clear outliers within the overall literature. Importantly, the authors of these studies themselves acknowledged that these results were unexpected and should be interpreted with caution. These isolated findings were not consistently replicated across independent cohorts or developmental stages and may reflect methodological differences, residual confounding, chance findings, or limited subgroup sample sizes.

Interpretation of the available evidence is limited by substantial heterogeneity across studies. Exposure assessment varied considerably across studies, with many relying on maternal self-report, potentially introducing recall bias and underreporting. Moreover, cannabis use during pregnancy frequently co-occurs with tobacco exposure, alcohol use, socioeconomic adversity, and maternal psychiatric conditions, each independently associated with adverse neurodevelopmental outcomes [10, 11]. While most studies adjusted for key confounders, attenuation of associations after comprehensive adjustment was common, particularly within cognitive domains. Residual confounding, including genetic, familial and environmental factors, cannot be excluded and may partially account for some observed associations. In addition, persistent cannabis use during pregnancy may, in some cases, reflect maternal self-medication for underlying psychiatric or neurodevelopmental conditions, including ADHD, anxiety, or mood disorders. These maternal conditions may independently contribute to offspring neurodevelopmental outcomes through genetic transmission, shared environmental influences, or altered parent-child interactions, thereby complicating causal interpretation of prenatal cannabis exposure effects.

Comparison with previous literature indicates general concordance regarding behavioural and attention-related vulnerabilities following prenatal cannabis exposure. However, earlier reviews have often aggregated maternal, perinatal, and child outcomes or combined prenatal and postnatal exposures, limiting the ability to isolate intrauterine effects. By organising findings according to neurodevelopmental domains and developmental stages, the present review provides a more differentiated synthesis, suggesting that associations appear concentrated in attentional, regulatory, and behavioural domains rather than reflecting uniform neurodevelopmental impairment.

### Strengths and limitations

This review has several strengths. It provides a comprehensive domain-specific synthesis of neurodevelopmental outcomes across a broad developmental range, incorporates structured risk-of-bias assessment, and considers the impact of confounder adjustment when interpreting study findings. The inclusion of both longitudinal cohorts and large population-based datasets further enhances the generalisability of the evidence.

Nevertheless, several limitations should be acknowledged. All included studies were observational in design, which precludes causal inference. The heterogeneity of findings across studies likely reflects important methodological differences. In particular, exposure assessment represented a major source of variability, as several studies relied primarily on maternal self-report, whereas others used biological measures such as urine or meconium testing. Underreporting of cannabis use during pregnancy may therefore have contributed to exposure misclassification and attenuation of observed associations. In addition, neurodevelopmental outcomes were assessed using a wide range of instruments and statistical metrics, further limiting comparability across studies and the possibility of a formal quantitative meta-analysis. Finally, certain domains, particularly executive functioning and visuospatial processing, were represented by relatively few studies, reducing the precision of domain-specific conclusions.

### Clinical and public health implications

Although current evidence does not consistently demonstrate global cognitive or language impairment following prenatal cannabis exposure, the recurrent associations observed in behavioural regulation, attention, and executive functioning warrant careful consideration. Even modest alterations in these domains may have downstream consequences for academic achievement, social functioning, and long-term mental health trajectories. In addition, prenatal cannabis exposure has been associated with increased risks of adverse obstetric and neonatal outcomes, including preterm birth, low birth weight, small-for-gestational-age status, and neonatal intensive care unit admission [5–7].

Given the increasing social acceptance of cannabis use and the growing misperception of its safety, healthcare professionals should provide clear, balanced, and evidence-informed counselling during pregnancy. Moreover, the potency of cannabis products, particularly Δ9-tetrahydrocannabinol (THC) concentration, has increased substantially in recent years, raising further concern regarding potential foetal exposure levels. Strengthening public health education and clinical guidance is therefore essential to address misconceptions surrounding prenatal cannabis use and to reduce potentially preventable adverse outcomes [2, 94]. Particular attention may be warranted for behavioural regulation, attention, and executive functioning, which appear to show greater vulnerability in the available literature. Prevention strategies should focus on early identification of prenatal cannabis exposure and the implementation of supportive interventions targeting these domains in at-risk children. Future research should prioritise well-designed prospective studies with precise quantification of exposure, trimester-specific analyses, and rigorous control for familial, genetic, psychiatric, and environmental confounding factors, including parental neurodevelopmental and mental health conditions. Such approaches are essential to clarify potential causal pathways, identify sensitive developmental windows, and determine whether observed associations follow a true dose-response pattern.

## Conclusion

In summary, the available evidence does not consistently support a uniform association between prenatal cannabis exposure and global neurodevelopmental impairment. Instead, findings suggest a more selective pattern of vulnerability, with behavioural regulation, attention, and executive functioning emerging as the domains most consistently associated with exposure, while global cognition, language, visuospatial abilities, and early motor development appear less consistently affected after adjustment for confounders. This pattern aligns with neurodevelopmental models proposing that higher-order regulatory networks may be more vulnerable to prenatal environmental perturbations than early global developmental indices.

Some evidence further suggests that the magnitude of these associations may depend on both the intensity and timing of exposure, with heavier or persistent use, particularly beyond maternal awareness of pregnancy, more frequently associated with adverse outcomes. These findings raise the possibility that risk may not simply reflect the presence of exposure, but may also depend on dose-response and developmental timing mechanisms. However, substantial methodological heterogeneity and the possibility of residual confounding limit causal interpretation. These patterns may also partially reflect residual familial, psychiatric, and genetic confounding, particularly in relation to persistent maternal cannabis use during pregnancy.

From a clinical perspective, these findings support cautious, evidence-informed counselling regarding cannabis use during pregnancy and highlight the need for continued high-quality longitudinal research with improved exposure and outcome measurements and rigorous control of familial, genetic and environmental confounding. As cannabis use during pregnancy continues to increase in many regions, improving the evidence base surrounding its potential neurodevelopmental consequences remains a critical priority for both clinical practice and public health.

## Supplementary Information

Below is the link to the electronic supplementary material.

Supplementary Material 1 (DOCX 273 KB)

## Data Availability

All data supporting the findings of this study are available within the paper and its Supplementary Information.

## References

[1] Young-Wolff KC, Sarovar V, Tucker LY, Avalos LA, Alexeeff S, Conway A et al (2019) Trends in marijuana use among pregnant women with and without nausea and vomiting in pregnancy, 2009–2016. Drug Alcohol Depend 196:66–70. 10.1016/j.drugalcdep.2018.12.009 PMC6392046 30711893

[2] ACOG Clinical Consensus No (2025) 10: Cannabis Use During Pregnancy and Lactation. Obstet Gynecol 146(4):600–611. 10.1097/aog.0000000000006053 40966737

[3] Grigsby TJ, Assoumou B, Koning SM, Howard JT, Howard K (2025) Cannabis use among pregnant and nonpregnant women of childbearing age: findings from the 2021–2023 National Survey of Drug Use and Health. Am J Prev Med 69(4):107967. 10.1016/j.amepre.2025.107967 40614880

[4] First OK, MacGibbon KW, Cahill CM, Cooper ZD, Gelberg L, Cortessis VK et al (2022) Patterns of Use and Self-reported Effectiveness of Cannabis for Hyperemesis Gravidarum. Geburtshilfe Frauenheilkd 82(5):517–527. 10.1055/a-1749-5391 PMC9076215 35528189

[5] Lo JO, Ayers CK, Yeddala S, Shaw B, Robalino S, Ward R et al (2025) Prenatal Cannabis Use and Neonatal Outcomes: A Systematic Review and Meta-Analysis. JAMA Pediatr 179(7):738–746. 10.1001/jamapediatrics.2025.0689 PMC12053797 40323610

[6] Luke S, Hobbs AJ, Smith M, Riddell C, Murphy P, Agborsangaya C et al (2022) Cannabis use in pregnancy and maternal and infant outcomes: A Canadian cross-jurisdictional population-based cohort study. PLoS ONE 17(11):e0276824. 10.1371/journal.pone.0276824 PMC9683571 36417349

[7] Marchand G, Masoud AT, Govindan M, Ware K, King A, Ruther S et al (2022) Birth Outcomes of Neonates Exposed to Marijuana in Utero: A Systematic Review and Meta-analysis. JAMA Netw Open 5(1):e2145653. 10.1001/jamanetworkopen.2021.45653 PMC8796018 35084479

[8] Huizink AC (2014) Prenatal cannabis exposure and infant outcomes: overview of studies. Prog Neuropsychopharmacol Biol Psychiatry 52:45–52. 10.1016/j.pnpbp.2013.09.014 24075896

[9] Grant KS, Petroff R, Isoherranen N, Stella N, Burbacher TM (2018) Cannabis use during pregnancy: Pharmacokinetics and effects on child development. Pharmacol Ther 182:133–151. 10.1016/j.pharmthera.2017.08.014 PMC6211194 28847562

[10] Sorkhou M, Singla DR, Castle DJ, George TP (2024) Birth, cognitive and behavioral effects of intrauterine cannabis exposure in infants and children: A systematic review and meta-analysis. Addiction 119(3):411–437. 10.1111/add.16370 PMC10872597 37968824

[11] Smid M, Metz T, McMillin G, Mele L, Casey B, Reddy U et al (2022) Prenatal Nicotine or Cannabis Exposure and Offspring Neurobehavioral Outcomes. Obstet Gynecol 139(1):21–30. 10.1097/AOG.0000000000004632 PMC8715943 34856574

[12] Kumar AR, Benson LS, Wymore EM, Phipers JE, Dempsey JC, Cort LA et al (2025) Quantification and prediction of human fetal (-)-∆(9)-tetrahydrocannabinol/(±)-11-OH-∆(9)-tetrahydrocannabinol exposure during pregnancy to inform fetal cannabis toxicity. Nat Commun 16(1):824. 10.1038/s41467-025-55863-5 PMC11743202 39827121

[13] Kumar AR, Sheikh ED, Monson JW, Ligon SE, Talley RL, Dornisch EM et al (2023) Understanding the Mechanism and Extent of Transplacental Transfer of (-)-∆(9) -Tetrahydrocannabinol (THC) in the Perfused Human Placenta to Predict In Vivo Fetal THC Exposure. Clin Pharmacol Ther 114(2):446–458. 10.1002/cpt.2964 37278090

[14] Richardson KA, Hester AK, McLemore GL (2016) Prenatal cannabis exposure - The first hit to the endocannabinoid system. Neurotoxicol Teratol 58:5–14. 10.1016/j.ntt.2016.08.003 27567698

[15] DeVuono MV, Nashed MG, Sarikahya MH, Kocsis A, Lee K, Vanin SR et al (2024) Prenatal tetrahydrocannabinol and cannabidiol exposure produce sex-specific pathophysiological phenotypes in the adolescent prefrontal cortex and hippocampus. Neurobiol Dis 199:106588. 10.1016/j.nbd.2024.106588 38960101

[16] Sarikahya MH, Cousineau SL, De Felice M, Szkudlarek HJ, Wong KKW, DeVuono MV et al (2023) Prenatal THC exposure induces long-term, sex-dependent cognitive dysfunction associated with lipidomic and neuronal pathology in the prefrontal cortex-hippocampal network. Mol Psychiatry 28(10):4234–4250. 10.1038/s41380-023-02190-0 37525013

[17] Thomason ME, Palopoli AC, Jariwala NN, Werchan DM, Chen A, Adhikari S et al (2021) Miswiring the brain: Human prenatal ∆9-tetrahydrocannabinol use associated with altered fetal hippocampal brain network connectivity. Dev Cogn Neurosci 51:101000. 10.1016/j.dcn.2021.101000 PMC8363827 34388638

[18] Sujan AC, Young-Wolff KC, Avalos LA (2022) In-utero cannabis exposure and long-term psychiatric and neurodevelopmental outcomes: The limitations of existing literature and recommendations for future research. Birth Defects Res 114(13):689–713. 10.1002/bdr2.2060 PMC9357094 35708102

[19] Page MJ, McKenzie JE, Bossuyt PM, Boutron I, Hoffmann TC, Mulrow CD et al (2021) The PRISMA 2020 statement: an updated guideline for reporting systematic reviews. BMJ 372:n71. 10.1136/bmj.n71 PMC8005924 33782057

[20] Schardt C, Adams MB, Owens T, Keitz S, Fontelo P (2007) Utilization of the PICO framework to improve searching PubMed for clinical questions. BMC Med Inf Decis Mak 7:16. 10.1186/1472-6947-7-16 PMC1904193 17573961

[21] Wells GA, Wells G, Shea B, Shea B, O’Connell D, Peterson J et al (eds) The Newcastle-Ottawa Scale (NOS) for Assessing the Quality of Nonrandomised Studies in Meta-Analyses2014

[22] Williams K, Thomson D, Seto I, Contopoulos-Ioannidis DG, Ioannidis JP, Curtis S et al (2012) Standard 6: age groups for pediatric trials. Pediatrics 129(Suppl 3):S153–S160. 10.1542/peds.2012-0055I 22661762

[23] Fried PA (1989) Postnatal consequences of maternal marijuana use in humans. Ann N Y Acad Sci 562:123–132. 10.1111/j.1749-6632.1989.tb21011.x 2742270

[24] Richardson G, Day N, Goldschmidt L (1995) Prenatal alcohol, marijuana, and tobacco use: infant mental and motor development. Neurotoxicol Teratol 17(4):479–87 10.1016/0892-0362(95)00006-d 7565494

[25] Talavera-Barber M, Morehead E, Ziegler K, Hockett C, Elliott A (2023) Prenatal cannabinoid exposure and early language development. Front Pead 11:1290707. 10.3389/fped.2023.1290707 PMC10702953 38078314

[26] Tennes K, Avitable N, Blackard C, Boyles C, Hassoun B, Holmes L et al (1985) Marijuana: prenatal and postnatal exposure in the human. NIDA Res Monogr 59:48–60. 3929132

[27] Jacobson S, Chiodo L, Sokol R, Jacobson J (2002) Validity of maternal report of prenatal alcohol, cocaine, and smoking in relation to neurobehavioral outcome. Pediatrics 109(5):815–825. 10.1542/peds.109.5.815 11986441

[28] Serino MD, Peterson MB, Rosen MT (2018) Psychological Functioning of Women Taking Illicit Drugs during Pregnancy and the Growth and Development of Their Offspring in Early Childhood. J dual diagnosis 14(3):158–170. 10.1080/15504263.2018.1468946 PMC6202263 29694295

[29] Singer L, Arendt R, Farkas K, Minnes S, Huang J, Yamashita T (1997) Relationship of prenatal cocaine exposure and maternal postpartum psychological distress to child developmental outcome. Dev Psychopathol 9(3):473–489. 10.1017/s0954579497001259 PMC4213812 9327234

[30] Fried P, Watkinson B (1988) 12- and 24-month neurobehavioural follow-up of children prenatally exposed to marihuana, cigarettes and alcohol. Neurotoxicol Teratol 10(4):305–313. 10.1016/0892-0362(88)90032-3 3226373

[31] Pleau J, Tanguay N, Courtemanche Y, Séguin J, Herba C, Simard M et al (2025) Longitudinal Associations Between Cannabis Use during Pregnancy and Child Cognitive, Motor, and Language Development at 2 Years Old. Matern Child Health J 29(4):549–562. 10.1007/s10995-025-04077-8 40121379

[32] Fried PA, The Ottawa Prenatal Prospective Study (OPPS) (1995) Methodological issues and findings: It’s easy to throw the baby out with the bath water. Life Sci 56(23):2159–2168. 10.1016/0024-3205(95)00203-I 7539879

[33] Day N, Richardson G, Goldschmidt L, Robles N, Taylor P, Stoffer D et al (1994) Effect of prenatal marijuana exposure on the cognitive development of offspring at age three. Neurotoxicol Teratol 16(2):169–175. 10.1016/0892-0362(94)90114-7 8052191

[34] Griffith D, Azuma S, Chasnoff I (1994) Three-year outcome of children exposed prenatally to drugs. J Am Acad Child Adolesc Psychiatry 33(1):20–27. 10.1097/00004583-199401000-00004 7511139

[35] Jarque P, Carmona M, Roca A, Barcelo B, Pichini S, Elorza M et al (2024) Evaluation of three-year neurodevelopmental outcomes in infants prenatally exposed to substance use. Drug Alcohol Depend 259:111284. 10.1016/j.drugalcdep.2024.111284 38640866

[36] Hayes J, Lampart R, Dreher M, Morgan L (1991) Five-year follow-up of rural Jamaican children whose mothers used marijuana during pregnancy. West Indian Med J 40(3):120–123. 1957518

[37] Moore B, Salmons K, Hoyt A, Swenson K, Bates E, Sauder K et al (2023) Associations between prenatal and postnatal exposure to cannabis with cognition and behavior at age 5 years: the Healthy Start Study. Int J Environ Res Public Health. 10.3390/ijerph20064880 PMC10049128 36981794

[38] Fried PA, O’Connell CM, Watkinson B (1992) 60- and 72-month follow-up of children prenatally exposed to marijuana, cigarettes, and alcohol: Cognitive and language assessment. J Dev Behav Pediatr 13(6):383–391. 10.1097/00004703-199212000-00001 1469105

[39] Bennett D, Bendersky M, Lewis M (2008) Children’s cognitive ability from 4 to 9 years old as a function of prenatal cocaine exposure, environmental risk, and maternal verbal intelligence. Dev Psychol 44(4):919–928. 10.1037/0012-1649.44.4.919 PMC2556289 18605824

[40] Corsi D, Donelle J, Sucha E, Hawken S, Hsu H, El-Chaâr D et al (2020) Maternal cannabis use in pregnancy and child neurodevelopmental outcomes. Nat Med 26(10):1536–1540. 10.1038/s41591-020-1002-5 32778828

[41] Goldschmidt L, Richardson G, Willford J, Day N (2008) Prenatal marijuana exposure and intelligence test performance at age 6. J Am Acad Child Adolesc Psychiatry 47(3):254–263. 10.1097/CHI.0b013e318160b3f0 18216735

[42] O’Connell CH Prenatal exposure to cannabis: An investigation of postnatal consequences in school age children 1992 10.1016/0892-0362(91)90047-z 1779951

[43] Crone S The effects of prenatal cannabis exposure on offspring’s behavior, cognition, developmental process, and executive functioning: An archival data examination 2022

[44] Cioffredi L, Anderson H, Loso H, East J, Nguyen P, Garavan H et al (2022) Prenatal cannabis exposure predicts attention problems, without changes on fMRI in adolescents. Neurotoxicol Teratol 91:107089 10.1016/j.ntt.2022.107089 PMC9136933 35314358

[45] Richardson GA, Ryan C, Willford J, Day NL, Goldschmidt L (2002) Prenatal alcohol and marijuana exposure: Effects on neuropsychological outcomes at 10 years. Neurotoxicol Teratol 24(3):311–320. 10.1016/S0892-0362(02)00193-9 12009486

[46] Day N, Leech S, Goldschmidt L (2011) The effects of prenatal marijuana exposure on delinquent behaviors are mediated by measures of neurocognitive functioning. Neurotoxicol Teratol 33(1):129–136. 10.1016/j.ntt.2010.07.006 PMC3052937 21256427

[47] Goldschmidt L, Richardson G, Cornelius M, Day N Prenatal marijuana and alcohol exposure and academic achievement at age 10. United States2004 2004–7. 521–532 p 10.1016/j.ntt.2004.04.003 15203174

[48] Rose-Jacobs R, Augustyn M, Beeghly M, Martin B, Cabral HJ, Heeren TC et al (2012) Intrauterine substance exposures and Wechsler Individual Achievement Test-II scores at 11 years of age. Vulnerable Child Youth Stud 7(2):186–197. 10.1080/17450128.2011.648967

[49] Fried P, Watkinson B, Gray R (1998) Differential effects on cognitive functioning in 9- to 12-year olds prenatally exposed to cigarettes and marihuana. Neurotoxicol Teratol 20(3):293–306. 10.1016/S0892-0362(97)00091-3 9638687

[50] Fried PA, Watkinson B (2000) Visuoperceptual functioning differs in 9- to 12-year olds prenatally exposed to cigarettes and marihuana. Neurotoxicol Teratol 22(1):11–20. 10.1016/S0892-0362(99)00046-X 10642110

[51] Corsi D, Morris T, Reed Z, Davey SG (2025) Maternal cannabis use in pregnancy, perinatal outcomes, and cognitive development in offspring: a longitudinal analysis of the ALSPAC cohort using paternal cannabis use as a negative control exposure. Eur J Epidemiol 40(5):549–562. 10.1007/s10654-025-01233-w PMC12170737 40353977

[52] Fried PA, Watkinson B, Gray R (2003) Differential effects on cognitive functioning in 13- to 16-year olds prenatally exposed to cigarettes and marihuana. Neurotoxicol Teratol 25(4):427–436. 10.1016/S0892-0362(03)00029-1 12798960

[53] Goldschmidt L, Richardson G, Willford J, Severtson S, Day N (2012) School achievement in 14-year-old youths prenatally exposed to marijuana. Neurotoxicol Teratol 34(1):161–167. 10.1016/j.ntt.2011.08.009 PMC3260381 21884785

[54] Lester B, Tronick E, LaGasse L, Seifer R, Bauer C, Shankaran S et al The maternal lifestyle study: effects of substance exposure during pregnancy on neurodevelopmental outcome in 1-month-old infants. United States2002 2002–12. 1182–92 p 10.1542/peds.110.6.1182 12456917

[55] Peterson B, Rosen T, Dingman S, Toth Z, Sawardekar S, Hao X et al (2020) Associations of Maternal Prenatal Drug Abuse With Measures of Newborn Brain Structure, Tissue Organization, and Metabolite Concentrations. JAMA Pediatr 174(9):831–842. 10.1001/jamapediatrics.2020.1622 PMC7296459 32539126

[56] Faden V, Graubard B (2000) Maternal substance use during pregnancy and developmental outcome at age three. J Subst Abuse 12(4):329–340. 10.1016/s0899-3289(01)00052-9 11452837

[57] Murnan AW, Keim SA, Yeates KO, Boone KM, Sheppard KW, Klebanoff MA (2021) Behavioral and cognitive differences in early childhood related to prenatal marijuana exposure. J Appl Dev Psychol. 10.1016/j.appdev.2021.101348 PMC8622818 34840377

[58] Accornero V, Morrow C, Bandstra E, Johnson A, Anthony J (2002) Behavioral outcome of preschoolers exposed prenatally to cocaine: role of maternal behavioral health. J Pediatr Psychol 27(3):259–269. 10.1093/jpepsy/27.3.259 PMC2760334 11909933

[59] DiGuiseppi C, Crume T, Van Dyke J, Sabourin K, Soke G, Croen L et al (2022) Peri-Pregnancy Cannabis Use and Autism Spectrum Disorder in the Offspring: Findings from the Study to Explore Early Development. J Autism Dev Disord 52(11):5064–5071. 10.1007/s10803-021-05339-4 PMC9112286 34767135

[60] Chatterji P, Markowitz S (2001) The impact of maternal alcohol and illicit drug use on children’s behavior problems: evidence from the children of the national longitudinal survey of youth. J Health Econ 20(5):703–731. 10.1016/s0167-6296(01)00090-x 11558645

[61] El Marroun H, Bolhuis K, Franken I, Jaddoe V, Hillegers M, Lahey B et al (2019) Preconception and prenatal cannabis use and the risk of behavioural and emotional problems in the offspring; a multi-informant prospective longitudinal study. Int J Epidemiol 48(1):287–296. 10.1093/ije/dyy186 30239742

[62] Winiger E, Hewitt J (2020) Prenatal cannabis exposure and sleep outcomes in children 9–10 years of age in the adolescent brain cognitive development (SM) study. Sleep health 6(6):787–789. 10.1016/j.sleh.2020.05.006 PMC7749837 32605891

[63] Roffman J, Sipahi E, Dowling K, Hughes D, Hopkinson C, Lee H et al (2021) Association of adverse prenatal exposure burden with child psychopathology in the Adolescent Brain Cognitive Development (ABCD) Study. PLoS ONE 16(4):e0250235. 10.1371/journal.pone.0250235 PMC8081164 33909652

[64] Gu Z, Barch D, Luo Q (2024) Prenatal substance exposure and child health: Understanding the role of environmental factors, genetics, and brain development. PNAS nexus 3(1):pgae003. 10.1093/pnasnexus/pgae003 PMC10826906 38292554

[65] Gray K, Day N, Leech S, Richardson G Prenatal marijuana exposure: effect on child depressive symptoms at ten years of age. United States2005 2005–5. 439–48 p 10.1016/j.ntt.2005.03.010 15869861

[66] Goldschmidt L, Day N, Richardson G Effects of prenatal marijuana exposure on child behavior problems at age 10. United States2000 2000–5. 325–36 p 10.1016/s0892-0362(00)00066-0 10840176

[67] Vishnubhotla R, Ahmad S, Zhao Y, Radhakrishnan R (2024) Impact of prenatal marijuana exposure on adolescent brain structural and functional connectivity and behavioural outcomes. Brain Commun. 10.1093/braincomms/fcae001 PMC10914455 38444906

[68] Paul S, Hatoum A, Fine J, Johnson E, Hansen I, Karcher N et al (2021) Associations Between Prenatal Cannabis Exposure and Childhood Outcomes Results From the ABCD Study. JAMA PSYCHIATRY 78(1):64–76. 10.1001/jamapsychiatry.2020.2902 PMC7512132 32965490

[69] Nadler E, Jacobus J, Rabin R (2025) Prenatal Cannabis and Tobacco Co-Exposure and Its Association with Behavioural Outcomes in Middle Childhood: Co-exposition prénatale au cannabis et au tabac et son association avec les résultats comportementaux au cours de l’enfance intermédiaire. Can J psychiatry Revue canadienne de psychiatrie 70(1):41–53. 10.1177/07067437241271696 PMC11572036 39140868

[70] Baranger D, Paul S, Colbert S, Karcher N, Johnson E, Hatoum A et al (2022) Association of Mental Health Burden With Prenatal Cannabis Exposure From Childhood to Early Adolescence: Longitudinal Findings From the Adolescent Brain Cognitive Development (ABCD) Study. JAMA Pediatr 176(12):1261–1265. 10.1001/jamapediatrics.2022.3191 PMC9468940 36094599

[71] Tadesse A, Ayano G, Dachew B, Betts K, Alati R (2024) Exposure to maternal cannabis use disorder and risk of autism spectrum disorder in offspring: A data linkage cohort study. Psychiatry Res 337:115971. 10.1016/j.psychres.2024.115971 38788554

[72] Tadesse A, Dachew B, Ayano G, Betts K, Alati R (2025) Maternal cannabis use disorder and offspring behavioral outcomes: findings from a linked data cohort study. Psychiatry Res 346:116404. 10.1016/j.psychres.2025.116404 39956029

[73] Nutor C, Dickerson A, Hsu T, Al-Jadiri A, Camargo C, Schweitzer J et al (2024) Examining the association between prenatal cannabis exposure and child autism traits: A multi-cohort investigation in the environmental influences on child health outcome program. Autism Res 17(8):1651–1664. 10.1002/aur.3185 PMC11341247 38953698

[74] Salzwedel A, Chen G, Chen Y, Grewen K, Gao W (2020) Functional dissection of prenatal drug effects on baby brain and behavioral development. Hum Brain Mapp 41(17):4789–4803. 10.1002/hbm.25158 PMC7643353 32779835

[75] Watts D, Lebel C, Chaput K, Giesbrecht G, Dewsnap K, Baglot S et al (2024) Evaluation of the Association Between Prenatal Cannabis Use and Risk of Developmental Delay. JAACAP OPEN 2(4):250–262. 10.1016/j.jaacop.2024.03.004 PMC11650681 39697398

[76] Fried P, Watkinson B (1990) 36- and 48-month neurobehavioral follow-up of children prenatally exposed to marijuana, cigarettes, and alcohol. J Dev Behav pediatrics: JDBP 11(2):49–58. 2324288

[77] Avalos L, Oberman N, Alexeeff S, Croen L, Davignon M, Adams S et al (2024) Early Maternal Prenatal Cannabis Use and Child Developmental Delays. JAMA Netw open 7(10):e2440295. 10.1001/jamanetworkopen.2024.40295 PMC11581621 39422907

[78] Fried PA, Watkinson B, Siegel LS (1997) Reading and language in 9- to 12- year olds prenatally exposed to cigarettes and marijuana. Neurotoxicol Teratol 19(3):171–183. 10.1016/S0892-0362(97)00015-9 9200137

[79] Noland J, Singer L, Short E, Minnes S, Arendt R, Kirchner H et al (2005) Prenatal drug exposure and selective attention in preschoolers. Neurotoxicol Teratol 27(3):429–438. 10.1016/j.ntt.2005.02.001 15939203

[80] Fried PA, Watkinson B, Gray R (1992) A follow-up study of attentional behavior in 6-year-old children exposed prenatally to marijuana, cigarettes, and alcohol. Neurotoxicol Teratol 14(5):299–311. 10.1016/0892-0362(92)90036-A 1454038

[81] Young-Wolff K, Kong K, Alexeeff S, Croen L, Oberman N, Kirane H et al (2025) Prenatal Cannabis Use and Offspring Attention Deficit Hyperactivity Disorder and Disruptive Behavior Disorders: A Retrospective Cohort Study. J Dev Behav pediatrics: JDBP 46(1):e25–e32. 10.1097/DBP.0000000000001323 PMC11832326 39400201

[82] Ryan JE, McCabe SE, Wilens TE, Weigard A, Worster B, Veliz P (2024) Prenatal polysubstance use and attention-deficit/hyperactivity disorder (ADHD). Am J Prev Med 66(3):548–550. 10.1016/j.amepre.2023.11.023 PMC10922843 38070629

[83] Fried PA, Watkinson B (2001) Differential effects on facets of attention in adolescents prenatally exposed to cigarettes and marihuana. Neurotoxicol Teratol 23(5):421–430. 10.1016/S0892-0362(01)00160-X 11711244

[84] Tchuente V, Sheehy O, Zhao J, Gorgui J, Gomez Y, Berard A (2022) Is in-utero exposure to cannabis associated with the risk of attention deficit with or without hyperactivity disorder? A cohort study within the Quebec Pregnancy Cohort. BMJ open 12(8):e052220. 10.1136/bmjopen-2021-052220 PMC9364390 35940828

[85] Chandler L, Richardson G, Gallagher J, Day N (1996) Prenatal exposure to alcohol and marijuana: effects on motor development of preschool children. Alcoholism, clinical and experimental research. 20(3):455–461. 10.1111/j.1530-0277.1996.tb01075.x 8727237

[86] Noland J, Singer L, Arendt R, Minnes S, Short E, Bearer C Executive functioning in preschool-age children prenatally exposed to alcohol, cocaine, and marijuana. England2003 2003–4. 647–56 p 10.1097/01.ALC.0000060525.10536.F6 PMC2597170 12711927

[87] Leech S, Richardson G, Goldschmidt L, Day N Prenatal substance exposure: effects on attention and impulsivity of 6-year-olds. United States1999 1999–3. 109–18 p 10.1016/s0892-0362(98)00042-7 10192271

[88] Rose-Jacobs R, Soenksen S, Appugliese D, Cabral H, Richardson M, Beeghly M et al (2011) Early adolescent executive functioning, intrauterine exposures and own drug use. Neurotoxicol Teratol 33(3):379–392. 10.1016/j.ntt.2011.02.013 PMC3145371 21371553

[89] Rose-Jacobs R, Richardson M, Buchanan-Howland K, Chen C, Cabral H, Heeren T et al (2017) Intrauterine exposure to tobacco and executive functioning in high school. Drug Alcohol Depend 176:169–175. 10.1016/j.drugalcdep.2017.02.022 PMC5539953 28544995

[90] Bailey B, Osborne J (2023) Prenatal marijuana exposure and visual perception in toddlers: Evidence of a sensory processing deficit. Front Pead 11:1113047. 10.3389/fped.2023.1113047 PMC10017869 36937971

[91] Chakraborty A, Anstice N, Jacobs R, LaGasse L, Lester B, Wouldes T et al (2015) Prenatal exposure to recreational drugs affects global motion perception in preschool children. Sci Rep 5:16921. 10.1038/srep16921 PMC4652269 26581958

[92] Hiraoka D, Makita K, Hamatani S, Tomoda A, Mizuno Y (2023) Effects of prenatal cannabis exposure on developmental trajectory of cognitive ability and brain volumes in the adolescent brain cognitive development (ABCD) study. Dev Cogn Neurosci 60:101209. 10.1016/j.dcn.2023.101209 PMC9950823 36791556

[93] Baron Nelson M, O’Neil SH, Wisnowski JL, Hart D, Sawardekar S, Rauh V et al (2019) Maturation of Brain Microstructure and Metabolism Associates with Increased Capacity for Self-Regulation during the Transition from Childhood to Adolescence. J Neurosci 39(42):8362–8375. 10.1523/jneurosci.2422-18.2019 PMC6794926 31444243

[94] Andrade C (2024) Maternal cannabis use during pregnancy and maternal and neonatal adverse outcomes. J Clin Psychiatry. 10.4088/JCP.24f15611 39480147

