# Supplementary material for: Neurodevelopmental outcomes following prenatal cannabinoid exposure: a systematic review
Source: Eur Child Adolesc Psychiatry. 2026 Jul 4;35(10):3073–98. doi: 10.1007/s00787-026-03126-z (PMC13696604; doi:10.1007/s00787-026-03126-z)
Supplement: Supplementary file 1 [file 787_2026_3126_MOESM1_ESM.docx]

**Title:** Neurodevelopmental outcomes following prenatal cannabinoid exposure: a systematic review

**Authors:** Maria Leonor Domingues^1^*, Bruno Miguel Fonseca^2,3#^, Susana Isabel Sá^1,4#^

**Affiliations:** ^1^ Department of Biomedicine, Faculty of Medicine, University of Porto, Alameda Prof. Hernâni Monteiro, 4200-319, Porto, Portugal. ^2^UCIBIO – Applied Molecular Biosciences Unit, Laboratório de Bioquímica, Faculdade de Farmácia, Universidade do Porto, Rua de Jorge Viterbo Ferreira 228, 4050-313 Porto, Portugal. ^3^Associate Laboratory i4HB - Institute for Health and Bioeconomy, Faculdade de Farmácia, Universidade do Porto, 4050-313 Porto, Portugal. ^4^RISE-Health, Faculty of Medicine, University of Porto, Alameda Prof. Hernâni Monteiro, 4200-319, Porto, Portugal.

*Corresponding author: [leonorddomingues@gmail.com](mailto:leonorddomingues@gmail.com)

^#^Co-senior authors

**Table S1:** Database Search Strategy

| **Database** | Pubmed via Medline |
| --- | --- |
| **Search query** | ("Cannabis"[Mesh] OR "Cannabinoids"[Mesh] OR cannabis[tiab] OR marijuana[tiab] OR cannabinoid*[tiab] OR THC[tiab] OR tetrahydrocannabinol[tiab] OR cannabidiol[tiab]) AND ("Pregnancy"[Mesh] OR "Prenatal"[Mesh] OR "Maternal Exposure"[Mesh] OR pregnancy[tiab] OR pregnant[tiab] OR prenatal[tiab] OR antenatal[tiab] OR perinatal[tiab] OR postnatal[tiab] OR maternal[tiab] OR "in utero"[tiab]) AND ("Child Development"[Mesh] OR "Neurodevelopmental Disorders"[Mesh] OR "Neuropsychological Tests"[Mesh] OR "Intelligence Tests"[Mesh] OR "Bayley Scales"[Mesh] OR "Cognition"[Mesh] OR neurodevelopment*[tiab] OR neurocognitive[tiab] OR cognitive[tiab] OR intelligence[tiab] OR "intelligence quotient"[tiab] OR IQ[tiab] OR Bayley[tiab] OR "Bayley Scales"[tiab] OR WISC[tiab] OR WPPSI[tiab] OR Wechsler[tiab] OR "developmental delay"[tiab] OR "cognitive function"[tiab]) AND ("Infant"[Mesh] OR "Child"[Mesh] OR "Child, Preschool"[Mesh] OR "Adolescent"[Mesh] OR infant*[tiab] OR newborn*[tiab] OR neonat*[tiab] OR toddler*[tiab] OR child*[tiab] OR adolescen*[tiab]) |
| **Database last searched** | 25/10/2025 |
| **Articles yielded** | 557 |

| **Database** | Web of Science |
| --- | --- |
| **Search query** | TS=(cannabis OR marijuana OR cannabinoid* OR THC OR tetrahydrocannabinol OR cannabidiol) AND TS=(pregnancy OR pregnant OR prenatal OR antenatal OR perinatal OR postnatal OR maternal OR "in utero") AND TS=(neurodevelopment* OR neurocognitive OR cognitive OR "child development" OR "mental development" OR "intelligence quotient" OR IQ OR Bayley OR "Bayley Scales" OR WISC OR WPPSI OR Wechsler OR "developmental delay" OR "cognitive function") AND TS=(infant* OR newborn* OR neonat* OR toddler* OR child* OR children OR adolescen*) |
| **Database last searched** | 25/10/2025 |
| **Articles yielded** | 607 |

| **Database** | PsycINFO |
| --- | --- |
| **Search query** | TX (DE "Cannabis" OR DE "Marijuana" OR DE "Cannabinoids" OR cannabis OR marijuana OR cannabinoid* OR THC OR tetrahydrocannabinol OR cannabidiol) AND TX ((DE "Pregnancy" OR DE "Prenatal Influences" OR DE "Maternal Influences" OR pregnancy OR pregnant OR prenatal OR antenatal OR perinatal OR postnatal OR maternal OR "in utero")) AND TX (DE "Child Development" OR DE "Neurodevelopmental Disorders" OR DE "Cognitive Development" OR DE "Neuropsychological Tests" OR DE "Intelligence Tests" OR neurodevelopment* OR neurocognitive OR cognitive OR "child development" OR "mental development" OR "intelligence quotient" OR IQ OR Bayley OR "Bayley Scales" OR WISC OR WPPSI OR Wechsler OR "developmental delay" OR "cognitive function") AND TX ((DE "Infants" OR DE "Children" OR DE "Adolescents" OR infant* OR newborn* OR neonat* OR toddler* OR child* OR children OR adolescen*)) |
| **Database last searched** | 25/10/2025 |
| **Articles yielded** | 396 |

**Table S2:** Excluded Articles and Reasons for Exclusion

| **Article** | **Reasons for exclusion** |
| --- | --- |
| 1. Acosta-Rodriguez H, Bobba P, Zeevi T, Ment L, Payabvash S. The Effect of Prenatal Marijuana Exposure on White Matter Microstructure and Cortical Morphology during Late Childhood. AJNR American journal of neuroradiology. 2025. [1] | Outcome not relevant to the review question |
| 2. Astley S, Little R. Maternal marijuana use during lactation and infant development at one year. Neurotoxicology and teratology. 1990;12(2):161–8. [2] | Postnatal exposure only |
| 3. Bandstra E, Vogel A, Morrow C, Xue L, Anthony J. Severity of prenatal cocaine exposure and child language functioning through age seven years: a longitudinal latent growth curve analysis. Substance use & misuse. 2004;39(1):25–59. [3] | Cannabis exposure not analysed independently |
| 4. Bauer C. Perinatal effects of prenatal drug exposure - Neonatal aspects. CLINICS IN PERINATOLOGY. 1999;26(1):87. [4] | Book chapter |
| 5. Bertrand K, Borchelt J, Honerkamp-Smith G, Akshoomoff N, Chambers C. Infant Adverse Reactions, Postnatal Growth and Neurodevelopmental Outcomes in Infants and Toddlers Breastfed by Mothers Who Use Marijuana 2020. [5] | Conference abstract only |
| 6. Conerly RC. Prenatal drug exposure: An examination of cognitive, language, and social skills 1999. [6] | Cannabis exposure not analysed independently |
| 7. Conradt E, McGrath M, Knapp E, Li X, Musci R, Mansolf M, et al. Prenatal Substance Exposure: Associations with Neurodevelopment in Middle Childhood. AMERICAN JOURNAL OF PERINATOLOGY. 2024;41:e1944–e54. [7] | Cannabis exposure not analysed independently |
| 8. DiGuiseppi C, Crume T, Holst B, Aiona K, Van Dyke J, Croen L, et al. Associations of maternal peripregnancy cannabis use with behavioral and developmental outcomes in children with and without symptoms of autism spectrum disorder: Study to Explore Early Development. Autism research : official journal of the International Society for Autism Research. 2025;18(1):202–16. [8] | Inappropriate study design |
| 9. Dreher MC, Hayes JS. Triangulation in cross-cultural research of child development in Jamaica. Western Journal of Nursing Research. 1993;15(2):216–29. [9] | Inappropriate study design |
| 10. El Marroun H, Tiemeier H, Steegers EAP, Jaddoe VWV, Hofman A, Verhulst FC, et al. Intrauterine cannabis exposure affects fetal growth trajectories: The Generation R Study. Journal of the American Academy of Child &amp; Adolescent Psychiatry. 2009;48(12):1173–81. [10] | Outcome not relevant to the review question |
| 11. Fine JD, Moreau AL, Karcher NR, Agrawal A, Rogers CE, Barch DM, et al. Association of prenatal cannabis exposure with psychosis proneness among children in the adolescent brain cognitive development (ABCD) study. JAMA Psychiatry. 2019;76(7):762–4. [11] | Outcome not relevant to the review question |
| 12. Fried MW. Psychosocial and pharmacological predictors of the cognitive functioning of children prenatally exposed to illicit drugs 2005. [12] | Cannabis exposure not analysed independently |
| 13. Garrison-Desany H, Hong X, Maher B, Beaty T, Wang G, Pearson C, et al. Individual and Combined Association Between Prenatal Polysubstance Exposure and Childhood Risk of Attention-Deficit/Hyperactivity Disorder. JAMA network open. 2022;5(3):e221957. [13] | Inappropriate population |
| 14. Goncalves P, Woodruff J, Martins S, Albaugh M, Talati A. Prenatal cannabis exposure and cortical development trajetories across preadolescence- ages 9 to 13 years NEUROPSYCHOPHARMACOLOGY. 2024;49:262–3. [14] | Conference abstract only |
| 15. Kiblawi Z, Smith L, Diaz S, LaGasse L, Derauf C, Newman E, et al. Prenatal methamphetamine exposure and neonatal and infant neurobehavioral outcome: results from the IDEAL study. Substance abuse. 2014;35(1):68–73. [15] | Cannabis exposure not analysed independently |
| 16. Klebanoff M, Fried P, Yeates K, Rausch J, Wilkins D, Blei H, et al. Lifestyle and Early Achievement in Families (LEAF) study: Design of an ambidirectional cohort study of prenatal marijuana exposure and child development and behaviour. Paediatric and perinatal epidemiology. 2020;34(6):744–56. [16] | Protocol only |
| 17. Leech S, Larkby C, Day R, Day N. Predictors and correlates of high levels of depression and anxiety symptoms among children at age 10. Journal of the American Academy of Child and Adolescent Psychiatry. 2006;45(2):223–30. [17] | Outcome not relevant to the review question |
| 18. Lemvigh C, Brouwer R, Hilker R, Anhøj S, Baandrup L, Pantelis C, et al. The relative and interactive impact of multiple risk factors in schizophrenia spectrum disorders: a combined register-based and clinical twin study. Psychological medicine. 2023;53(4):1266–76. [18] | Inappropriate population |
| 19. Lewis B, Singer L, Short E, Minnes S, Arendt R, Weishampel P, et al. Four-year language outcomes of children exposed to cocaine in utero. United States2004 2004–9. 617–27 p. [19] | Cannabis exposure not analysed independently |
| 20. Maxwell J, Cioffredi L, Talavera-Barber M, Henry M, Beauman S, Hittson A, et al. The protocol for a pilot feasibility trial of Improving Neurodevelopmental ouTcomes After prenatal Cannabinoid in uTero exposure (INTACT) study for a multi-center trial. United States2025 2025. e0322035 p. [20] | Protocol only |
| 21. McLemore G, Richardson K. Data from three prospective longitudinal human cohorts of prenatal marijuana exposure and offspring outcomes from the fetal period through young adulthood. Data in brief. 2016;9:753–7. [21] | Inappropriate study design |
| 22. Min M, Minnes S, Park H, Ridenour T, Kim J, Yoon M, et al. Developmental trajectories of externalizing behavior from ages 4 to 12: Prenatal cocaine exposure and adolescent correlates. Drug and alcohol dependence. 2018;192:223–32. [22] | Outcome not relevant to the review question |
| 23. Monshouwer K, Huizink A, Harakeh Z, Raaijmakers Q, Reijneveld S, Oldehinkel A, et al. Prenatal smoking exposure and the risk of behavioral problems and substance use in adolescence: the TRAILS study. European addiction research. 2011;17(6):342–50. [23] | Inappropriate study design |
| 24. Moore DG, Turner JD, Parrott AC, Goodwin JE, Fulton SE, Min MO, et al. During pregnancy, recreational drug-using women stop taking ecstasy (3,4-methylenedioxy-N-methylamphetamine) and reduce alcohol consumption, but continue to smoke tobacco and cannabis: Initial findings from the Development and Infancy Study. Journal of Psychopharmacology. 2010;24(9):1403–10. [24] | Outcome not relevant to the review question |
| 25. O'Connor A, O'Brien L, Watson L. Implications of perinatal buprenorphine exposure on infant head circumference at birth. The journal of maternal-fetal & neonatal medicine : the official journal of the European Association of Perinatal Medicine, the Federation of Asia and Oceania Perinatal Societies, the International Society of Perinatal Obstetricians. 2021;34(2):318–22. [25] | Outcome not relevant to the review question |
| 26. Qiu M, Lanclos SL, Holland J, Torres CA. Prenatal drug exposure: Lead by language limitations. Drug and Alcohol Dependence. 2020;206. [26] | Letter to the editor |
| 27. Richardson G, Conroy M, Day N. Prenatal cocaine exposure: effects on the development of school-age children. United States1996 1996–11. 627–34 p. [27] | Cannabis exposure not analysed independently |
| 28. Rivkin M, Davis P, Lemaster J, Cabral H, Warfield S, Mulkern R, et al. Volumetric MRI study of brain in children with intrauterine exposure to cocaine, alcohol, tobacco, and marijuana. United States2008 2008–4. 741–50 p. [28] | Outcome not relevant to the review question |
| 29. Rosen TS, Johnson HL. Drug-addicted mothers, their infants, and SIDS. Annals of the New York Academy of Sciences. 1988;533:89–95. [29] | Cannabis exposure not analysed independently |
| 30. Roussos-Ross K, Dukharan V, Goodin A. Is In-Utero Marijuana Exposure Associated With Childhood Developmental Delay? OBSTETRICS AND GYNECOLOGY. 2020;135:118S–9S. [30] | Conference abstract only |
| 31. Shisler S, Lee J, Schlienz N, Hawk LJ, Thanos P, Kong K, et al. Prenatal tobacco and tobacco-cannabis co-exposure: Relationship with attention and memory in middle childhood. NEUROTOXICOLOGY AND TERATOLOGY. 2024;104. [31] | Cannabis exposure not analysed independently |
| 32. Singer L, Min M, Minnes S, Short E, Lewis B, Lang A, et al. Prenatal and concurrent cocaine, alcohol, marijuana, and tobacco effects on adolescent cognition and attention. Drug and alcohol dependence. 2018;191:37–44. [32] | Cannabis exposure not analysed independently |
| 33. Smid M, McMillin G, Metz T. Early prenatal marijuana and nicotine exposure and child neurodevelopmental outcomes. AMERICAN JOURNAL OF OBSTETRICS AND GYNECOLOGY. 2021;224(2):S38–S9. [33] | Duplicate article |
| 34. Smith A, Fried P, Hogan M, Cameron I. Effects of prenatal marijuana on visuospatial working memory: an fMRI study in young adults. United States2006 2006–3. 286–95 p. [34] | Inappropriate population |
| 35. Smith L, LaGasse L, Derauf C, Newman E, Shah R, Haning W, et al. Motor and cognitive outcomes through three years of age in children exposed to prenatal methamphetamine. Neurotoxicology and teratology. 2011;33(1):176–84. [35] | Cannabis exposure not analysed independently |
| 36. Spechler P, Thompson W, Paulus M. Prenatal Cannabis Exposure Moderates the Relationship Between Sleep Hours and Internalizing Problems: A Causal Inference Analysis of ABCD Data. BIOLOGICAL PSYCHIATRY. 2022;91(9):S94–S. [36] | Conference abstract only |
| 37. Spechler PA, Gutierrez RM, Tapert SF, Thompson WK, Paulus MP. The beneficial effect of sleep on behavioral health problems in youth is disrupted by prenatal cannabis exposure: A causal random forest analysis of Adolescent Brain Cognitive Development data. Child Development. 2023;94(4):826–35. [37] | Inappropriate study design |
| 38. Stevens H, Olsavsky A, Riggs P. Cannabis use across lifespan: implications for prenatal and adolescent neurodevelopment. Journal of the american academy of child and adolescent psychiatry. 2022;61(10):S277–S. [38] | Conference abstract only |
| 39. Stroud L, Papandonatos G, McCallum M, Kehoe T, Salisbury A, Huestis M. Prenatal tobacco and marijuana co-use: Impact on newborn neurobehavior. Neurotoxicology and teratology. 2018;70:28–39. [39] | Cannabis exposure not analysed independently |
| 40. Winiger E, Hewitt J. Prenatal cannabis use and sleep outcomes in children 9-10 years of age in the adolescence brain cognitive development study. SLEEP. 2020;43:A353–A4. [40] | Conference abstract only |

**Table S3:** Quality Assessment of Included Studies using the Newcastle-Ottawa-Scale

Note: Each cell represents one question of the Newcastle-Ottawa-Scale. A 🗸 indicates that the criterion in question was fulfilled. NOS Scores of 7–9 indicate high methodological quality, 5–6 indicate medium methodological quality, and 1–4 indicate low methodological quality.

| Autor, year | Selection | | | | Comparability | | Outcome | | | Total points |
| --- | --- | --- | --- | --- | --- | --- | --- | --- | --- | --- |
|  | 1 | 2 | 3 | 4 | 1a | 1b | 1 | 2 | 3 |  |
| Accornero *et al*., 2002 [41] | 🗸 | 🗸 | 🗸 | 🗸 | 🗸 | 🗸 | 🗸 | 🗸 | 🗸 | 9 |
| Avalos *et al*., 2024 [42] | 🗸 | 🗸 | 🗸 | 🗸 | 🗸 | 🗸 | 🗸 |  |  | 7 |
| Bailey *et al*., 2023 [43] | 🗸 | 🗸 | 🗸 | 🗸 | 🗸 | 🗸 | 🗸 | 🗸 | 🗸 | 9 |
| Baranger *et al*., 2022 [44] | 🗸 | 🗸 |  | 🗸 | 🗸 | 🗸 | 🗸 | 🗸 | 🗸 | 8 |
| Bennett *et al*., 2008 [45] | 🗸 | 🗸 | 🗸 | 🗸 | 🗸 | 🗸 | 🗸 | 🗸 |  | 8 |
| Chakraborty *et al*., 2015 [46] | 🗸 | 🗸 | 🗸 | 🗸 | 🗸 | 🗸 | 🗸 | 🗸 | 🗸 | 9 |
| Chandler *et al*., 1996 [47] | 🗸 | 🗸 |  | 🗸 | 🗸 | 🗸 | 🗸 | 🗸 | 🗸 | 8 |
| Chatterji *et al*., 2001 [48] | 🗸 | 🗸 |  | 🗸 | 🗸 | 🗸 | 🗸 | 🗸 | 🗸 | 8 |
| Cioffredi *et al*., 2022 [49] | 🗸 | 🗸 |  | 🗸 | 🗸 | 🗸 | 🗸 | 🗸 | 🗸 | 8 |
| Corsi *et al*., 2020 [50] | 🗸 | 🗸 |  | 🗸 | 🗸 | 🗸 | 🗸 | 🗸 | 🗸 | 8 |
| Corsi *et al*., 2025 [51] | 🗸 | 🗸 |  | 🗸 | 🗸 | 🗸 | 🗸 | 🗸 | 🗸 | 8 |
| Crone *et al*., 2022 [52] | 🗸 | 🗸 |  | 🗸 |  |  | 🗸 | 🗸 |  | 5 |
| Day *et al*., 1994 [53] | 🗸 | 🗸 |  | 🗸 | 🗸 | 🗸 | 🗸 | 🗸 | 🗸 | 8 |
| Day *et al*., 2011 [54] | 🗸 | 🗸 | 🗸 | 🗸 | 🗸 | 🗸 | 🗸 | 🗸 |  | 8 |
| DiGuiseppi *et al*., 2022 [55] | 🗸 | 🗸 |  | 🗸 | 🗸 | 🗸 | 🗸 | 🗸 | 🗸 | 8 |
| El Marroun *et al*., 2019 [56] | 🗸 | 🗸 | 🗸 | 🗸 | 🗸 | 🗸 | 🗸 | 🗸 | 🗸 | 9 |
| Faden *et al*., 2000 [57] | 🗸 | 🗸 |  | 🗸 | 🗸 | 🗸 |  | 🗸 |  | 6 |
| Fried *et al*., 1988 [58] | 🗸 | 🗸 |  | 🗸 | 🗸 | 🗸 | 🗸 | 🗸 | 🗸 | 8 |
| Fried *et al*., 1989 [59] | 🗸 | 🗸 |  | 🗸 | 🗸 | 🗸 | 🗸 | 🗸 | 🗸 | 8 |
| Fried *et al*., 1990 [60] | 🗸 | 🗸 |  | 🗸 | 🗸 | 🗸 | 🗸 | 🗸 | 🗸 | 8 |
| Fried *et al*., 1992 [61] | 🗸 | 🗸 |  | 🗸 | 🗸 | 🗸 | 🗸 | 🗸 | 🗸 | 8 |
| Fried *et al*., 1992 [62] | 🗸 | 🗸 |  | 🗸 | 🗸 | 🗸 | 🗸 | 🗸 | 🗸 | 8 |
| Fried *et al*., 1995 [63] | 🗸 | 🗸 |  | 🗸 | 🗸 | 🗸 | 🗸 | 🗸 | 🗸 | 8 |
| Fried *et al*., 1997 [64] | 🗸 | 🗸 |  | 🗸 | 🗸 | 🗸 | 🗸 | 🗸 | 🗸 | 8 |
| Fried *et al*., 1998 [65] | 🗸 | 🗸 |  | 🗸 | 🗸 | 🗸 | 🗸 | 🗸 | 🗸 | 8 |
| Fried *et al*., 2000 [66] | 🗸 | 🗸 |  | 🗸 | 🗸 | 🗸 | 🗸 | 🗸 | 🗸 | 8 |
| Fried *et al*., 2001 [67] | 🗸 | 🗸 |  | 🗸 | 🗸 | 🗸 | 🗸 | 🗸 | 🗸 | 8 |
| Fried *et al*., 2003 [68] | 🗸 | 🗸 |  | 🗸 | 🗸 | 🗸 | 🗸 | 🗸 | 🗸 | 8 |
| Goldschmidt *et al*., 2000 [69] | 🗸 | 🗸 |  | 🗸 | 🗸 | 🗸 | 🗸 | 🗸 | 🗸 | 8 |
| Goldschmidt *et al*., 2004 [70] | 🗸 | 🗸 |  | 🗸 | 🗸 | 🗸 | 🗸 | 🗸 | 🗸 | 8 |
| Goldschmidt *et al*., 2008 [71] | 🗸 | 🗸 |  | 🗸 | 🗸 | 🗸 | 🗸 | 🗸 | 🗸 | 8 |
| Goldschmidt *et al*., 2012 [72] | 🗸 | 🗸 |  | 🗸 | 🗸 | 🗸 | 🗸 | 🗸 | 🗸 | 8 |
| Gray *et al*., 2005 [73] | 🗸 | 🗸 |  | 🗸 | 🗸 | 🗸 | 🗸 | 🗸 | 🗸 | 8 |
| Griffith *et al*., 1994 [74] | 🗸 | 🗸 | 🗸 | 🗸 | 🗸 | 🗸 | 🗸 | 🗸 | 🗸 | 9 |
| Gu *et al*., 2024 [75] | 🗸 | 🗸 |  | 🗸 | 🗸 | 🗸 | 🗸 | 🗸 | 🗸 | 8 |
| Hayes *et al*., 1991 [76] | 🗸 | 🗸 | 🗸 | 🗸 | 🗸 | 🗸 | 🗸 | 🗸 | 🗸 | 9 |
| Hiraoka *et al*., 2023 [77] | 🗸 | 🗸 |  | 🗸 | 🗸 | 🗸 | 🗸 | 🗸 | 🗸 | 8 |
| Jacobson *et al*., 2002 [78] | 🗸 | 🗸 | 🗸 | 🗸 | 🗸 | 🗸 | 🗸 | 🗸 | 🗸 | 9 |
| Jarque *et al*., 2024 [79] | 🗸 | 🗸 | 🗸 | 🗸 | 🗸 |  | 🗸 | 🗸 | 🗸 | 8 |
| Leech *et al*., 1999 [80] | 🗸 | 🗸 |  | 🗸 | 🗸 | 🗸 | 🗸 | 🗸 | 🗸 | 8 |
| Lester *et al*., 2002 [81] |  | 🗸 | 🗸 | 🗸 | 🗸 | 🗸 | 🗸 | 🗸 | 🗸 | 8 |
| Moore *et al*., 2023 [82] |  | 🗸 | 🗸 | 🗸 | 🗸 | 🗸 | 🗸 | 🗸 |  | 7 |
| Murnan *et al*., 2021 [83] | 🗸 | 🗸 | 🗸 | 🗸 | 🗸 | 🗸 | 🗸 | 🗸 | 🗸 | 9 |
| Nadler *et al*., 2025 [84] | 🗸 | 🗸 |  | 🗸 | 🗸 | 🗸 | 🗸 | 🗸 | 🗸 | 8 |
| Noland *et al*., 2003 [85] | 🗸 | 🗸 | 🗸 | 🗸 | 🗸 | 🗸 | 🗸 | 🗸 | 🗸 | 9 |
| Noland *et al*., 2005 [86] | 🗸 | 🗸 | 🗸 | 🗸 | 🗸 | 🗸 | 🗸 | 🗸 | 🗸 | 9 |
| Nutor *et al*., 2024 [87] | 🗸 | 🗸 | 🗸 | 🗸 | 🗸 | 🗸 | 🗸 | 🗸 | 🗸 | 9 |
| O’Connel *et al*., 1992 [88] | 🗸 | 🗸 |  | 🗸 | 🗸 | 🗸 | 🗸 | 🗸 | 🗸 | 8 |
| Paul *et al*., 2021 [89] | 🗸 | 🗸 |  | 🗸 | 🗸 | 🗸 | 🗸 |  | 🗸 | 7 |
| Peterson *et al*., 2020 [90] | 🗸 | 🗸 | 🗸 | 🗸 | 🗸 | 🗸 | 🗸 | 🗸 |  | 8 |
| Pleau *et al*., 2025 [91] | 🗸 | 🗸 |  | 🗸 | 🗸 | 🗸 | 🗸 | 🗸 | 🗸 | 8 |
| Reece *et al*., 2023 [92] | 🗸 | 🗸 | 🗸 |  | 🗸 | 🗸 | 🗸 | 🗸 | 🗸 | 8 |
| Richardson *et al*., 1995 [93] | 🗸 | 🗸 | 🗸 |  | 🗸 | 🗸 | 🗸 | 🗸 | 🗸 | 8 |
| Richardson *et al*., 2002 [94] | 🗸 | 🗸 | 🗸 |  | 🗸 | 🗸 | 🗸 | 🗸 | 🗸 | 8 |
| Roffman *et al*., 2021 [95] | 🗸 | 🗸 |  | 🗸 | 🗸 | 🗸 | 🗸 | 🗸 | 🗸 | 8 |
| Rose-Jacobs *et al*., 2011 [96] | 🗸 | 🗸 | 🗸 | 🗸 | 🗸 | 🗸 | 🗸 | 🗸 | 🗸 | 9 |
| Rose-Jacobs *et al*., 2012 [97] | 🗸 | 🗸 | 🗸 | 🗸 | 🗸 | 🗸 | 🗸 | 🗸 | 🗸 | 9 |
| Rose-Jacobs *et al*., 2017 [98] | 🗸 | 🗸 | 🗸 | 🗸 | 🗸 | 🗸 | 🗸 | 🗸 | 🗸 | 9 |
| Ryan *et al*., 2024 [99] | 🗸 | 🗸 |  | 🗸 | 🗸 | 🗸 | 🗸 | 🗸 | 🗸 | 8 |
| Salzwedel *et al*., 2020 [100] | 🗸 | 🗸 | 🗸 | 🗸 | 🗸 | 🗸 | 🗸 | 🗸 |  | 8 |
| Serino *et al*., 2018 [101] | 🗸 | 🗸 | 🗸 | 🗸 | 🗸 | 🗸 | 🗸 | 🗸 |  | 8 |
| Singer *et al*., 1997 [102] | 🗸 | 🗸 | 🗸 | 🗸 | 🗸 | 🗸 | 🗸 | 🗸 |  | 8 |
| Smid *et al*., 2022 [103] | 🗸 | 🗸 | 🗸 | 🗸 | 🗸 | 🗸 | 🗸 | 🗸 | 🗸 | 9 |
| Tadesse *et al*., 2024 [104] | 🗸 | 🗸 | 🗸 | 🗸 | 🗸 | 🗸 | 🗸 | 🗸 | 🗸 | 9 |
| Tadesse *et al*., 2025 [105] | 🗸 | 🗸 | 🗸 | 🗸 | 🗸 | 🗸 | 🗸 | 🗸 | 🗸 | 9 |
| Talavera-Barber *et al*., 2023 [106] | 🗸 | 🗸 |  | 🗸 | 🗸 | 🗸 | 🗸 | 🗸 | 🗸 | 8 |
| Tchuente *et al*., 2022 [107] | 🗸 | 🗸 |  | 🗸 | 🗸 | 🗸 | 🗸 | 🗸 |  | 7 |
| Tennes *et al*., 1985 [108] | 🗸 | 🗸 |  | 🗸 | 🗸 | 🗸 | 🗸 | 🗸 |  | 7 |
| Vishnubhotla *et al*., 2024 [109] | 🗸 | 🗸 |  | 🗸 | 🗸 | 🗸 | 🗸 | 🗸 | 🗸 | 8 |
| Watts *et al*., 2024 [110] | 🗸 | 🗸 |  | 🗸 | 🗸 | 🗸 |  | 🗸 | 🗸 | 7 |
| Winiger *et al*., 2020 [111] | 🗸 | 🗸 |  | 🗸 | 🗸 | 🗸 | 🗸 | 🗸 | 🗸 | 8 |
| Young-Wolf *et al*., 2025 [112] | 🗸 | 🗸 | 🗸 | 🗸 | 🗸 | 🗸 | 🗸 |  | 🗸 | 8 |

**Table S4:** Main covariates adjusted for across included studies

| Autor, year | Main covariates ajusted for |
| --- | --- |
| Accornero *et al*., 2002 [41] | Child sex, child age, child IQ, caregiver factors, maternal age, maternal education, maternal employment |
| Avalos *et al*., 2024 [42] | Maternal age, race/ethnicity, maternal education, neighborhood deprivation, parity, socioeconomic factors, prenatal care initiation, tobacco exposure, alcohol use, co-substance use, maternal medical comorbidities, maternal mental health |
| Bailey *et al*., 2023 [43] | Maternal age, maternal education, socioeconomic factors, tobacco use |
| Baranger *et al*., 2022 [44] | Child age, child sex, race/ethnicity, household income, maternal education, pubertal status, family psychiatric/substance use history, tobacco exposure, alcohol use, child substance use |
| Bennett *et al*., 2008 [45] | Other substance exposure, alcohol use, tobacco exposure, home environment, maternal stress, maternal age, maternal verbal IQ, child sex, neonatal medical risk |
| Chakraborty *et al*., 2015 [46] | Tobacco use, other substance use, child sex, ethnicity, visual function, verbal IQ |
| Chandler *et al*., 1996 [47] | Child age, child anthropometric measures, child IQ, race/ethnicity, child sex, maternal income, family structure, prenatal and postnatal substance exposure |
| Chatterji *et al*., 2001 [48] | Child age, child sex, race/ethnicity, birth weight, maternal education, maternal cognitive ability, household income, public assistance, marital status changes, maternal employment, family structure, maternal mental health, tobacco exposure |
| Cioffredi *et al*., 2022 [49] | Parental psychopathology, maternal age, breastfeeding duration, prematurity, race/ethnicity, household education |
| Corsi *et al*., 2020 [50] | Maternal age, maternal education, maternal medical comorbidities, maternal mental health, other substance use, pregnancy complications, preterm birth |
| Corsi *et al*., 2025 [51] | Child sex, parity, maternal age, maternal BMI, tobacco exposure, alcohol use, other substance use, parental education, socioeconomic status, maternal mental health |
| Crone *et al*., 2022 [52] | Unadjusted analysis |
| Day *et al*., 1994 [53] | Race/ethnicity, maternal age, maternal education, marital status, household income, parity, maternal mental health, social support, child sex, preschool attendance, other substance exposure |
| Day *et al*., 2011 [54] | Race/ethnicity, child sex, alcohol use, tobacco exposure, maternal IQ, home environment, household income, maternal mental health |
| DiGuiseppi *et al*., 2022 [55] | Maternal education, tobacco exposure, alcohol use |
| El Marroun *et al*., 2019 [56] | Maternal age, ethnicity, maternal education, maternal BMI, alcohol use, maternal mental health, child age, child sex, birth weight, tobacco exposure |
| Faden *et al*., 2000 [57] | Birth weight, child age, child sex, race/ethnicity, parental education, marital status |
| Fried *et al*., 1988 [58] | Family income, maternal age, maternal education, maternal nutrition, parity, birth weight, gestational age, home environment |
| Fried *et al*., 1989 [59] | Birth weight, socioeconomic status, tobacco exposure, alcohol use, caffeine exposure, maternal weight gain, nutrition, maternal age |
| Fried *et al*., 1990 [60] | Family income, maternal age, maternal education, maternal weight and nutrition, tobacco exposure, alcohol use, parity, gestational age, birth weight, home environment |
| Fried *et al*., 1992 [61] | Family income, maternal weight factors, maternal age, parental education, child sex, home environment, tobacco exposure, alcohol use |
| Fried *et al*., 1992 [62] | Family income, parental education, maternal age, nutrition, parity, child sex, home environment, alcohol use, tobacco exposure |
| Fried *et al*., 1995 [63] | Socioeconomic status, maternal age, maternal health, maternal personality factors, home environment, tobacco exposure, alcohol use |
| Fried *et al*., 1997 [64] | Tobacco exposure, alcohol use, maternal weight gain, parental education, family income, home environment |
| Fried *et al*., 1998 [65] | Maternal age, tobacco exposure, alcohol use, family income, parental education, child sex, postnatal substance exposure |
| Fried *et al*., 2000 [66] | Tobacco exposure, alcohol use, parental education |
| Fried *et al*., 2001 [67] | Alcohol use, tobacco exposure, family income, maternal education, maternal age, child sex, adolescent tobacco use |
| Fried *et al*., 2003 [68] | Alcohol use, tobacco exposure, socioeconomic status, maternal age, passive smoke exposure, home environment, adolescent tobacco use |
| Goldschmidt *et al*., 2000 [69] | Maternal education, race/ethnicity, household income, maternal mental health, life events, home environment, family conflict, child hospitalizations, other substance exposure (alcohol, tobacco, cocaine), current maternal substance use |
| Goldschmidt *et al*., 2004 [70] | Maternal education, race/ethnicity, social support, employment status, home environment, family structure, other substance exposure, child mental health |
| Goldschmidt *et al*., 2008 [71] | Maternal cognitive ability, race/ethnicity, social support, home environment, household size, child medical factors, maternal mental health, partner alcohol use |
| Goldschmidt *et al*., 2012 [72] | Race/ethnicity, maternal education, child sex, family structure, family income, home environment |
| Gray *et al*., 2005 [73] | Child IQ, tobacco exposure, child hospitalizations, maternal education, home environment, life events |
| Griffith *et al*., 1994 [74] | Other substance exposure, alcohol use, tobacco exposure, head circumference, behavioural factors |
| Gu *et al*., 2024 [75] | Pregnancy-related factors, breastfeeding, other substance exposure, socioeconomic factors, parental age, neighborhood factors, family psychiatric history, genetic risk factors |
| Hayes *et al*., 1991 [76] | Home environment, school attendance, child age, parity, socioeconomic status |
| Hiraoka *et al*., 2023 [77] | Child sex, child age, race/ethnicity, birth weight, maternal age, maternal education, socioeconomic status, family psychiatric history, tobacco exposure, alcohol use, pregnancy-related factors |
| Jacobson *et al*., 2002 [78] | Maternal age, maternal education, parity, maternal mental health, socioeconomic status, home environment, maternal IQ, alcohol use, tobacco exposure, other substance exposure |
| Jarque *et al*., 2024 [79] | Matching for gestational age, birth weight, and child sex; no adjustment for tobacco exposure or socioeconomic status |
| Leech *et al*., 1999 [80] | Child sex, race/ethnicity, child age, child medical factors, child IQ, home environment, household structure, maternal employment status, life events, maternal mental health, maternal age, current maternal substance use |
| Lester *et al*., 2002 [81] | Socioeconomic status, study site, birth weight, alcohol use, tobacco exposure, cannabis exposure, binge drinking, other substance exposure |
| Moore *et al*., 2023 [82] | Maternal age, maternal education, race/ethnicity, maternal mental health, child sex, child age, tobacco exposure |
| Murnan *et al*., 2021 [83] | Race/ethnicity, child age, child sex, tobacco exposure, marital status, household income, caregiver executive functioning |
| Nadler *et al*., 2025 [84] | Child sex, birth weight, pregnancy-related factors, maternal age, alcohol use, maternal education, household income, parental behavioural factors |
| Noland *et al*., 2003 [85] | Maternal age, tobacco exposure, alcohol use, other substance exposure, current caregiver substance use, verbal IQ |
| Noland *et al*., 2005 [86] | Prenatal cocaine exposure, current caregiver cannabis use |
| Nutor *et al*., 2024 [87] | Child sex, race/ethnicity, birth year, maternal age, maternal education, tobacco exposure, alcohol use, illicit substance exposure |
| O’Connel *et al*., 1992 [88] | Tobacco exposure, alcohol use, maternal age, maternal personality factors, home environment |
| Paul *et al*., 2021 [89] | Household income, race/ethnicity, maternal education, maternal age, family psychiatric history, tobacco exposure, alcohol use, pregnancy-related factors, child substance use |
| Peterson *et al*., 2020 [90] | Postmenstrual age at MRI, child sex, tobacco exposure, alcohol use, maternal age, race/ethnicity, socioeconomic status, maternal mental health, prenatal stress, neonatal anthropometric measures |
| Pleau *et al*., 2025 [91] | Maternal age, maternal education, alcohol use, maternal mental health, child sex, child age, tobacco exposure |
| Reece *et al*., 2023 [92] | Socioeconomic status, race/ethnicity, tobacco exposure, alcohol use, other substance use, cannabis potency measures |
| Richardson *et al*., 1995 [93] | Child age, child sex, educational/employment status, infant weight, hospitalizations, maternal sociodemographic factors, alcohol use, tobacco exposure |
| Richardson *et al*., 2002 [94] | Race/ethnicity, child sex, family income, home environment, child anxiety, tobacco exposure, alcohol use |
| Roffman *et al*., 2021 [95] | Child age, child sex, race/ethnicity, caregiver marital status, household income, parental education, maternal age, neighborhood factors, family structure |
| Rose-Jacobs *et al*., 2011 [96] | Child IQ, child sex, prenatal cocaine exposure, alcohol use, tobacco exposure, adolescent substance use |
| Rose-Jacobs *et al*., 2012 [97] | Maternal education, maternal country of origin, child sex, caregiver factors, cocaine exposure, alcohol use, tobacco exposure |
| Rose-Jacobs *et al*., 2017 [98] | Tobacco exposure, cocaine exposure, alcohol use, maternal age, race/ethnicity, maternal education, maternal country of origin, child sex, birth weight, child IQ, environmental exposures, child age at assessment |
| Ryan *et al*., 2024 [99] | Follow-up age, child sex, race/ethnicity, household income, maternal age, parental education, preterm birth, parental substance use history, maternal mental health |
| Salzwedel *et al*., 2020 [100] | Child sex, gestational age, gestational age at scan, birth weight, maternal mental health |
| Serino *et al*., 2018 [101] | Gestational age, alcohol use, tobacco exposure, socioeconomic status |
| Singer *et al*., 1997 [102] | Gestational age, maternal verbal ability, maternal mental health, cocaine exposure, tobacco exposure, alcohol use, child age at testing |
| Smid *et al*., 2022 [103] | Child age, insurance status, race/ethnicity, maternal education |
| Tadesse *et al*., 2024 [104] | Socioeconomic status, parity, tobacco use, other substance use, maternal mental health, maternal medical comorbidities, child sex, birth factors |
| Tadesse *et al*., 2025 [105] | Tobacco use, alcohol use, socioeconomic status, child sex, birth plurality, maternal mental health |
| Talavera-Barber *et al*., 2023 [106] | Maternal age, race/ethnicity, maternal education, household income, alcohol use, tobacco exposure, maternal mental health, gestational age, child sex |
| Tchuente *et al*., 2022 [107] | Maternal age, maternal education, household income, marital status, area of residence, race/ethnicity, parity, maternal BMI, tobacco exposure, alcohol use, other substance exposure, caffeine exposure, maternal lifestyle factors, maternal medical comorbidities, maternal mental health |
| Tennes *et al*., 1985 [108] | Maternal age, parity, race/ethnicity, socioeconomic status, caffeine exposure, alcohol use, cocaine exposure, tobacco exposure |
| Vishnubhotla *et al*., 2024 [109] | Child age, child sex, maternal education, partner education |
| Watts *et al*., 2024 [110] | Maternal age, race/ethnicity, household income, relationship status, maternal mental health, alcohol use, tobacco exposure, COVID-19 infection status, child sex |
| Winiger *et al*., 2020 [111] | Tobacco exposure, alcohol use, maternal education, household income, marital status, race/ethnicity, child sex, child age |
| Young-Wolf *et al*., 2025 [112] | Maternal sociodemographic factors, neighborhood deprivation, other substance use, prenatal care initiation, maternal medical comorbidities, maternal mental health, maternal BMI |

**References**

1.	Acosta-Rodriguez H, Bobba P, Zeevi T, Ment L, Payabvash S. The Effect of Prenatal Marijuana Exposure on White Matter Microstructure and Cortical Morphology during Late Childhood. AJNR American journal of neuroradiology. 2025.<https://doi.org/doi:10.3174/ajnr.A8774>

2.	Astley S, Little R. Maternal marijuana use during lactation and infant development at one year. Neurotoxicology and teratology. 1990;12(2):161–8.<https://doi.org/doi:10.1016/0892-0362(90)90129-z>

3.	Bandstra E, Vogel A, Morrow C, Xue L, Anthony J. Severity of prenatal cocaine exposure and child language functioning through age seven years: a longitudinal latent growth curve analysis. Substance use & misuse. 2004;39(1):25–59.<https://doi.org/doi:10.1081/ja-120027765>

4.	Bauer C. Perinatal effects of prenatal drug exposure - Neonatal aspects. CLINICS IN PERINATOLOGY. 1999;26(1):87–+.<https://doi.org/doi:10.1016/S0095-5108(18)30074-5>

5.	Bertrand K, Borchelt J, Honerkamp-Smith G, Akshoomoff N, Chambers C. Infant Adverse Reactions, Postnatal Growth and Neurodevelopmental Outcomes in Infants and Toddlers Breastfed by Mothers Who Use Marijuana2020 2020–6. 876– p.

6.	Conerly RC. Prenatal drug exposure: An examination of cognitive, language, and social skills 1999.

7.	Conradt E, McGrath M, Knapp E, Li X, Musci R, Mansolf M, et al. Prenatal Substance Exposure: Associations with Neurodevelopment in Middle Childhood. AMERICAN JOURNAL OF PERINATOLOGY. 2024;41:e1944–e54.<https://doi.org/doi:10.1055/a-2090-5293>

8.	DiGuiseppi C, Crume T, Holst B, Aiona K, Van Dyke J, Croen L, et al. Associations of maternal peripregnancy cannabis use with behavioral and developmental outcomes in children with and without symptoms of autism spectrum disorder: Study to Explore Early Development. Autism research : official journal of the International Society for Autism Research. 2025;18(1):202–16.<https://doi.org/doi:10.1002/aur.3284>

9.	Dreher MC, Hayes JS. Triangulation in cross-cultural research of child development in Jamaica. Western Journal of Nursing Research. 1993;15(2):216–29.<https://doi.org/doi:10.1177/019394599301500206>

10.	El Marroun H, Tiemeier H, Steegers EAP, Jaddoe VWV, Hofman A, Verhulst FC, et al. Intrauterine cannabis exposure affects fetal growth trajectories: The Generation R Study. Journal of the American Academy of Child & Adolescent Psychiatry. 2009;48(12):1173–81.<https://doi.org/doi:10.1097/CHI.0b013e3181bfa8ee>

11.	Fine JD, Moreau AL, Karcher NR, Agrawal A, Rogers CE, Barch DM, et al. Association of prenatal cannabis exposure with psychosis proneness among children in the adolescent brain cognitive development (ABCD) study. JAMA Psychiatry. 2019;76(7):762–4.<https://doi.org/doi:10.1001/jamapsychiatry.2019.0076>

12.	Fried MW. Psychosocial and pharmacological predictors of the cognitive functioning of children prenatally exposed to illicit drugs 2005.

13.	Garrison-Desany H, Hong X, Maher B, Beaty T, Wang G, Pearson C, et al. Individual and Combined Association Between Prenatal Polysubstance Exposure and Childhood Risk of Attention-Deficit/Hyperactivity Disorder. JAMA network open. 2022;5(3):e221957.<https://doi.org/doi:10.1001/jamanetworkopen.2022.1957>

14.	Goncalves P, Woodruff J, Martins S, Albaugh M, Talati A. Prenatal cannabis exposure and cortical development trajetories across preadolescence- ages 9 to 13 years. NEUROPSYCHOPHARMACOLOGY. 2024;49:262–3.<https://doi.org/doi>:

15.	Kiblawi Z, Smith L, Diaz S, LaGasse L, Derauf C, Newman E, et al. Prenatal methamphetamine exposure and neonatal and infant neurobehavioral outcome: results from the IDEAL study. Substance abuse. 2014;35(1):68–73.<https://doi.org/doi:10.1080/08897077.2013.814614>

16.	Klebanoff M, Fried P, Yeates K, Rausch J, Wilkins D, Blei H, et al. Lifestyle and Early Achievement in Families (LEAF) study: Design of an ambidirectional cohort study of prenatal marijuana exposure and child development and behaviour. Paediatric and perinatal epidemiology. 2020;34(6):744–56.<https://doi.org/doi:10.1111/ppe.12693>

17.	Leech S, Larkby C, Day R, Day N. Predictors and correlates of high levels of depression and anxiety symptoms among children at age 10. Journal of the American Academy of Child and Adolescent Psychiatry. 2006;45(2):223–30.<https://doi.org/doi:10.1097/01.chi.0000184930.18552.4d>

18.	Lemvigh C, Brouwer R, Hilker R, Anhøj S, Baandrup L, Pantelis C, et al. The relative and interactive impact of multiple risk factors in schizophrenia spectrum disorders: a combined register-based and clinical twin study. Psychological medicine. 2023;53(4):1266–76.<https://doi.org/doi:10.1017/S0033291721002749>

19.	Lewis B, Singer L, Short E, Minnes S, Arendt R, Weishampel P, et al. Four-year language outcomes of children exposed to cocaine in utero. United States2004 2004–9. 617–27 p.

20.	Maxwell J, Cioffredi L, Talavera-Barber M, Henry M, Beauman S, Hittson A, et al. The protocol for a pilot feasibility trial of Improving Neurodevelopmental ouTcomes After prenatal Cannabinoid in uTero exposure (INTACT) study for a multi-center trial. United States2025 2025. e0322035 p.

21.	McLemore G, Richardson K. Data from three prospective longitudinal human cohorts of prenatal marijuana exposure and offspring outcomes from the fetal period through young adulthood. Data in brief. 2016;9:753–7.<https://doi.org/doi:10.1016/j.dib.2016.10.005>

22.	Min M, Minnes S, Park H, Ridenour T, Kim J, Yoon M, et al. Developmental trajectories of externalizing behavior from ages 4 to 12: Prenatal cocaine exposure and adolescent correlates. Drug and alcohol dependence. 2018;192:223–32.<https://doi.org/doi:10.1016/j.drugalcdep.2018.08.007>

23.	Monshouwer K, Huizink A, Harakeh Z, Raaijmakers Q, Reijneveld S, Oldehinkel A, et al. Prenatal smoking exposure and the risk of behavioral problems and substance use in adolescence: the TRAILS study. European addiction research. 2011;17(6):342–50.<https://doi.org/doi:10.1159/000334507>

24.	Moore DG, Turner JD, Parrott AC, Goodwin JE, Fulton SE, Min MO, et al. During pregnancy, recreational drug-using women stop taking ecstasy (3,4-methylenedioxy-N-methylamphetamine) and reduce alcohol consumption, but continue to smoke tobacco and cannabis: Initial findings from the Development and Infancy Study. Journal of Psychopharmacology. 2010;24(9):1403–10.<https://doi.org/doi:10.1177/0269881109348165>

25.	O'Connor A, O'Brien L, Watson L. Implications of perinatal buprenorphine exposure on infant head circumference at birth. The journal of maternal-fetal & neonatal medicine : the official journal of the European Association of Perinatal Medicine, the Federation of Asia and Oceania Perinatal Societies, the International Society of Perinatal Obstetricians. 2021;34(2):318–22.<https://doi.org/doi:10.1080/14767058.2019.1599352>

26.	Qiu M, Lanclos SL, Holland J, Torres CA. Prenatal drug exposure: Lead by language limitations. Drug and Alcohol Dependence. 2020;206.<https://doi.org/doi:10.1016/j.drugalcdep.2019.107775>

27.	Richardson G, Conroy M, Day N. Prenatal cocaine exposure: effects on the development of school-age children. United States1996 1996–11. 627–34 p.

28.	Rivkin M, Davis P, Lemaster J, Cabral H, Warfield S, Mulkern R, et al. Volumetric MRI study of brain in children with intrauterine exposure to cocaine, alcohol, tobacco, and marijuana. United States2008 2008–4. 741–50 p.

29.	Rosen TS, Johnson HL. Drug-addicted mothers, their infants, and SIDS. Annals of the New York Academy of Sciences. 1988;533:89–95.<https://doi.org/doi:10.1111/j.1749-6632.1988.tb37236.x>

30.	Roussos-Ross K, Dukharan V, Goodin A. Is In-Utero Marijuana Exposure Associated With Childhood Developmental Delay? OBSTETRICS AND GYNECOLOGY. 2020;135:118S–9S.<https://doi.org/doi>:

31.	Shisler S, Lee J, Schlienz N, Hawk LJ, Thanos P, Kong K, et al. Prenatal tobacco and tobacco-cannabis co-exposure: Relationship with attention and memory in middle childhood. NEUROTOXICOLOGY AND TERATOLOGY. 2024;104.<https://doi.org/doi:10.1016/j.ntt.2024.107371>

32.	Singer L, Min M, Minnes S, Short E, Lewis B, Lang A, et al. Prenatal and concurrent cocaine, alcohol, marijuana, and tobacco effects on adolescent cognition and attention. Drug and alcohol dependence. 2018;191:37–44.<https://doi.org/doi:10.1016/j.drugalcdep.2018.06.022>

33.	Smid M, McMillin G, Metz T. Early prenatal marijuana and nicotine exposure and child neurodevelopmental outcomes. AMERICAN JOURNAL OF OBSTETRICS AND GYNECOLOGY. 2021;224(2):S38–S9.<https://doi.org/doi>:

34.	Smith A, Fried P, Hogan M, Cameron I. Effects of prenatal marijuana on visuospatial working memory: an fMRI study in young adults. United States2006 2006–3. 286–95 p.

35.	Smith L, LaGasse L, Derauf C, Newman E, Shah R, Haning W, et al. Motor and cognitive outcomes through three years of age in children exposed to prenatal methamphetamine. Neurotoxicology and teratology. 2011;33(1):176–84.<https://doi.org/doi:10.1016/j.ntt.2010.10.004>

36.	Spechler P, Thompson W, Paulus M. Prenatal Cannabis Exposure Moderates the Relationship Between Sleep Hours and Internalizing Problems: A Causal Inference Analysis of ABCD Data. BIOLOGICAL PSYCHIATRY. 2022;91(9):S94–S.<https://doi.org/doi>:

37.	Spechler PA, Gutierrez RM, Tapert SF, Thompson WK, Paulus MP. The beneficial effect of sleep on behavioral health problems in youth is disrupted by prenatal cannabis exposure: A causal random forest analysis of Adolescent Brain Cognitive Development data. Child Development. 2023;94(4):826–35.<https://doi.org/doi:10.1111/cdev.13899>

38.	Stevens H, Olsavsky A, Riggs P. Cannabis use across lifespan: implications for prenatal and adolescent neurodevelopment. Journal of the american academy of child and adolescent psychiatry. 2022;61(10):S277–S.<https://doi.org/doi:10.1016/j.jaac.2022.07.558>

39.	Stroud L, Papandonatos G, McCallum M, Kehoe T, Salisbury A, Huestis M. Prenatal tobacco and marijuana co-use: Impact on newborn neurobehavior. Neurotoxicology and teratology. 2018;70:28–39.<https://doi.org/doi:10.1016/j.ntt.2018.09.003>

40.	Winiger E, Hewitt J. Prenatal cannabis use and sleep outcomes in children 9-10 years of age in the adolescence brain cognitive development study. SLEEP. 2020;43:A353–A4.<https://doi.org/doi>:

41.	Accornero V, Morrow C, Bandstra E, Johnson A, Anthony J. Behavioral outcome of preschoolers exposed prenatally to cocaine: role of maternal behavioral health. Journal of pediatric psychology. 2002;27(3):259–69.<https://doi.org/doi:10.1093/jpepsy/27.3.259>

42.	Avalos L, Oberman N, Alexeeff S, Croen L, Davignon M, Adams S, et al. Early Maternal Prenatal Cannabis Use and Child Developmental Delays. JAMA network open. 2024;7(10):e2440295.<https://doi.org/doi:10.1001/jamanetworkopen.2024.40295>

43.	Bailey B, Osborne J. Prenatal marijuana exposure and visual perception in toddlers: Evidence of a sensory processing deficit. Frontiers in pediatrics. 2023;11:1113047.<https://doi.org/doi:10.3389/fped.2023.1113047>

44.	Baranger D, Paul S, Colbert S, Karcher N, Johnson E, Hatoum A, et al. Association of Mental Health Burden With Prenatal Cannabis Exposure From Childhood to Early Adolescence: Longitudinal Findings From the Adolescent Brain Cognitive Development (ABCD) Study. JAMA pediatrics. 2022;176(12):1261–5.<https://doi.org/doi:10.1001/jamapediatrics.2022.3191>

45.	Bennett D, Bendersky M, Lewis M. Children's cognitive ability from 4 to 9 years old as a function of prenatal cocaine exposure, environmental risk, and maternal verbal intelligence. Developmental psychology. 2008;44(4):919–28.<https://doi.org/doi:10.1037/0012-1649.44.4.919>

46.	Chakraborty A, Anstice N, Jacobs R, LaGasse L, Lester B, Wouldes T, et al. Prenatal exposure to recreational drugs affects global motion perception in preschool children. Scientific reports. 2015;5:16921.<https://doi.org/doi:10.1038/srep16921>

47.	Chandler L, Richardson G, Gallagher J, Day N. Prenatal exposure to alcohol and marijuana: effects on motor development of preschool children. Alcoholism, clinical and experimental research. 1996;20(3):455–61.<https://doi.org/doi:10.1111/j.1530-0277.1996.tb01075.x>

48.	Chatterji P, Markowitz S. The impact of maternal alcohol and illicit drug use on children's behavior problems: evidence from the children of the national longitudinal survey of youth. Journal of health economics. 2001;20(5):703–31.<https://doi.org/doi:10.1016/s0167-6296(01)00090-x>

49.	Cioffredi L, Anderson H, Loso H, East J, Nguyen P, Garavan H, et al. Prenatal cannabis exposure predicts attention problems, without changes on fMRI in adolescents. Neurotoxicology and teratology. 2022;91:107089.<https://doi.org/doi:10.1016/j.ntt.2022.107089>

50.	Corsi D, Donelle J, Sucha E, Hawken S, Hsu H, El-Chaâr D, et al. Maternal cannabis use in pregnancy and child neurodevelopmental outcomes. Nature medicine. 2020;26(10):1536–40.<https://doi.org/doi:10.1038/s41591-020-1002-5>

51.	Corsi D, Morris T, Reed Z, Davey SG. Maternal cannabis use in pregnancy, perinatal outcomes, and cognitive development in offspring: a longitudinal analysis of the ALSPAC cohort using paternal cannabis use as a negative control exposure. European journal of epidemiology. 2025;40(5):549–62.<https://doi.org/doi:10.1007/s10654-025-01233-w>

52.	Crone S. The effects of prenatal cannabis exposure on offspring’s behavior, cognition, developmental process, and executive functioning: An archival data examination 2022.

53.	Day N, Richardson G, Goldschmidt L, Robles N, Taylor P, Stoffer D, et al. Effect of prenatal marijuana exposure on the cognitive development of offspring at age three. Neurotoxicology and teratology. 1994;16(2):169–75.<https://doi.org/doi:10.1016/0892-0362(94)90114-7>

54.	Day N, Leech S, Goldschmidt L. The effects of prenatal marijuana exposure on delinquent behaviors are mediated by measures of neurocognitive functioning. Neurotoxicology and teratology. 2011;33(1):129–36.<https://doi.org/doi:10.1016/j.ntt.2010.07.006>

55.	DiGuiseppi C, Crume T, Van Dyke J, Sabourin K, Soke G, Croen L, et al. Peri-Pregnancy Cannabis Use and Autism Spectrum Disorder in the Offspring: Findings from the Study to Explore Early Development. Journal of autism and developmental disorders. 2022;52(11):5064–71.<https://doi.org/doi:10.1007/s10803-021-05339-4>

56.	El Marroun H, Bolhuis K, Franken I, Jaddoe V, Hillegers M, Lahey B, et al. Preconception and prenatal cannabis use and the risk of behavioural and emotional problems in the offspring; a multi-informant prospective longitudinal study. International journal of epidemiology. 2019;48(1):287–96.<https://doi.org/doi:10.1093/ije/dyy186>

57.	Faden V, Graubard B. Maternal substance use during pregnancy and developmental outcome at age three. Journal of substance abuse. 2000;12(4):329–40.<https://doi.org/doi:10.1016/s0899-3289(01)00052-9>

58.	Fried P, Watkinson B. 12- and 24-month neurobehavioural follow-up of children prenatally exposed to marihuana, cigarettes and alcohol. Neurotoxicology and teratology. 1988;10(4):305–13.<https://doi.org/doi:10.1016/0892-0362(88)90032-3>

59.	Fried PA. Postnatal consequences of maternal marijuana use in humans. Annals of the New York Academy of Sciences. 1989;562:123–32.<https://doi.org/doi:10.1111/j.1749-6632.1989.tb21011.x>

60.	Fried P, Watkinson B. 36- and 48-month neurobehavioral follow-up of children prenatally exposed to marijuana, cigarettes, and alcohol. Journal of developmental and behavioral pediatrics : JDBP. 1990;11(2):49–58.<https://doi.org/doi>:

61.	Fried PA, O'Connell CM, Watkinson B. 60- and 72-month follow-up of children prenatally exposed to marijuana, cigarettes, and alcohol: Cognitive and language assessment. Journal of Developmental and Behavioral Pediatrics. 1992;13(6):383–91.<https://doi.org/doi:10.1097/00004703-199212000-00001>

62.	Fried PA, Watkinson B, Gray R. A follow-up study of attentional behavior in 6-year-old children exposed prenatally to marijuana, cigarettes, and alcohol. Neurotoxicology and Teratology. 1992;14(5):299–311.<https://doi.org/doi:10.1016/0892-0362(92)90036-A>

63.	Fried PA. The Ottawa Prenatal Prospective Study (OPPS): Methodological issues and findings: It's easy to throw the baby out with the bath water. Life Sciences. 1995;56(23):2159–68.<https://doi.org/doi:10.1016/0024-3205(95)00203-I>

64.	Fried PA, Watkinson B, Siegel LS. Reading and language in 9- to 12- year olds prenatally exposed to cigarettes and marijuana. Neurotoxicology and Teratology. 1997;19(3):171–83.<https://doi.org/doi:10.1016/S0892-0362(97)00015-9>

65.	Fried P, Watkinson B, Gray R. Differential effects on cognitive functioning in 9- to 12-year olds prenatally exposed to cigarettes and marihuana. NEUROTOXICOLOGY AND TERATOLOGY. 1998;20(3):293–306.<https://doi.org/doi:10.1016/S0892-0362(97)00091-3>

66.	Fried PA, Watkinson B. Visuoperceptual functioning differs in 9- to 12-year olds prenatally exposed to cigarettes and marihuana. Neurotoxicology and Teratology. 2000;22(1):11–20.<https://doi.org/doi:10.1016/S0892-0362(99)00046-X>

67.	Fried PA, Watkinson B. Differential effects on facets of attention in adolescents prenatally exposed to cigarettes and marihuana. Neurotoxicology and Teratology. 2001;23(5):421–30.<https://doi.org/doi:10.1016/S0892-0362(01)00160-X>

68.	Fried PA, Watkinson B, Gray R. Differential effects on cognitive functioning in 13- to 16-year olds prenatally exposed to cigarettes and marihuana. Neurotoxicology and Teratology. 2003;25(4):427–36.<https://doi.org/doi:10.1016/S0892-0362(03)00029-1>

69.	Goldschmidt L, Day N, Richardson G. Effects of prenatal marijuana exposure on child behavior problems at age 10. United States2000 2000–5. 325–36 p.

70.	Goldschmidt L, Richardson G, Cornelius M, Day N. Prenatal marijuana and alcohol exposure and academic achievement at age 10. United States2004 2004–7. 521–32 p.

71.	Goldschmidt L, Richardson G, Willford J, Day N. Prenatal marijuana exposure and intelligence test performance at age 6. Journal of the American Academy of Child and Adolescent Psychiatry. 2008;47(3):254–63.<https://doi.org/doi:10.1097/CHI.0b013e318160b3f0>

72.	Goldschmidt L, Richardson G, Willford J, Severtson S, Day N. School achievement in 14-year-old youths prenatally exposed to marijuana. Neurotoxicology and teratology. 2012;34(1):161–7.<https://doi.org/doi:10.1016/j.ntt.2011.08.009>

73.	Gray K, Day N, Leech S, Richardson G. Prenatal marijuana exposure: effect on child depressive symptoms at ten years of age. United States2005 2005–5. 439–48 p.

74.	Griffith D, Azuma S, Chasnoff I. Three-year outcome of children exposed prenatally to drugs. Journal of the American Academy of Child and Adolescent Psychiatry. 1994;33(1):20–7.<https://doi.org/doi:10.1097/00004583-199401000-00004>

75.	Gu Z, Barch D, Luo Q. Prenatal substance exposure and child health: Understanding the role of environmental factors, genetics, and brain development. PNAS nexus. 2024;3(1):pgae003.<https://doi.org/doi:10.1093/pnasnexus/pgae003>

76.	Hayes J, Lampart R, Dreher M, Morgan L. Five-year follow-up of rural Jamaican children whose mothers used marijuana during pregnancy. The West Indian medical journal. 1991;40(3):120–3.<https://doi.org/doi>:

77.	Hiraoka D, Makita K, Hamatani S, Tomoda A, Mizuno Y. Effects of prenatal cannabis exposure on developmental trajectory of cognitive ability and brain volumes in the adolescent brain cognitive development (ABCD) study. Developmental cognitive neuroscience. 2023;60:101209.<https://doi.org/doi:10.1016/j.dcn.2023.101209>

78.	Jacobson S, Chiodo L, Sokol R, Jacobson J. Validity of maternal report of prenatal alcohol, cocaine, and smoking in relation to neurobehavioral outcome. Pediatrics. 2002;109(5):815–25.<https://doi.org/doi:10.1542/peds.109.5.815>

79.	Jarque P, Carmona M, Roca A, Barcelo B, Pichini S, Elorza M, et al. Evaluation of three-year neurodevelopmental outcomes in infants prenatally exposed to substance use. Drug and alcohol dependence. 2024;259:111284.<https://doi.org/doi:10.1016/j.drugalcdep.2024.111284>

80.	Leech S, Richardson G, Goldschmidt L, Day N. Prenatal substance exposure: effects on attention and impulsivity of 6-year-olds. United States1999 1999–3. 109–18 p.

81.	Lester B, Tronick E, LaGasse L, Seifer R, Bauer C, Shankaran S, et al. The maternal lifestyle study: effects of substance exposure during pregnancy on neurodevelopmental outcome in 1-month-old infants. United States2002 2002–12. 1182–92 p.

82.	Moore B, Salmons K, Hoyt A, Swenson K, Bates E, Sauder K, et al. Associations between Prenatal and Postnatal Exposure to Cannabis with Cognition and Behavior at Age 5 Years: The Healthy Start Study. International journal of environmental research and public health. 2023;20(6).<https://doi.org/doi:10.3390/ijerph20064880>

83.	Murnan AW, Keim SA, Yeates KO, Boone KM, Sheppard KW, Klebanoff MA. Behavioral and cognitive differences in early childhood related to prenatal marijuana exposure. Journal of Applied Developmental Psychology. 2021;77.<https://doi.org/doi:10.1016/j.appdev.2021.101348>

84.	Nadler E, Jacobus J, Rabin R. Prenatal Cannabis and Tobacco Co-Exposure and Its Association with Behavioural Outcomes in Middle Childhood: Co-exposition prénatale au cannabis et au tabac et son association avec les résultats comportementaux au cours de l'enfance intermédiaire. Canadian journal of psychiatry Revue canadienne de psychiatrie. 2025;70(1):41–53.<https://doi.org/doi:10.1177/07067437241271696>

85.	Noland J, Singer L, Arendt R, Minnes S, Short E, Bearer C. Executive functioning in preschool-age children prenatally exposed to alcohol, cocaine, and marijuana. England2003 2003–4. 647–56 p.

86.	Noland J, Singer L, Short E, Minnes S, Arendt R, Kirchner H, et al. Prenatal drug exposure and selective attention in preschoolers. Neurotoxicology and teratology. 2005;27(3):429–38.<https://doi.org/doi:10.1016/j.ntt.2005.02.001>

87.	Nutor C, Dickerson A, Hsu T, Al-Jadiri A, Camargo C, Schweitzer J, et al. Examining the association between prenatal cannabis exposure and child autism traits: A multi-cohort investigation in the environmental influences on child health outcome program. AUTISM RESEARCH. 2024;17(8):1651–64.<https://doi.org/doi:10.1002/aur.3185>

88.	O'Connell CH. Prenatal exposure to cannabis: An investigation of postnatal consequences in school age children 1992.

89.	Paul S, Hatoum A, Fine J, Johnson E, Hansen I, Karcher N, et al. Associations Between Prenatal Cannabis Exposure and Childhood Outcomes Results From the ABCD Study. JAMA PSYCHIATRY. 2021;78(1):64–76.<https://doi.org/doi:10.1001/jamapsychiatry.2020.2902>

90.	Peterson B, Rosen T, Dingman S, Toth Z, Sawardekar S, Hao X, et al. Associations of Maternal Prenatal Drug Abuse With Measures of Newborn Brain Structure, Tissue Organization, and Metabolite Concentrations. JAMA PEDIATRICS. 2020;174(9):831–42.<https://doi.org/doi:10.1001/jamapediatrics.2020.1622>

91.	Pleau J, Tanguay N, Courtemanche Y, Séguin J, Herba C, Simard M, et al. Longitudinal Associations Between Cannabis Use during Pregnancy and Child Cognitive, Motor, and Language Development at 2 Years Old. Maternal and child health journal. 2025;29(4):549–62.<https://doi.org/doi:10.1007/s10995-025-04077-8>

92.	Reece AS, Hulse GK. Impact of converging sociocultural and substance-related trends on US autism rates: Combined geospatiotemporal and causal inferential analysis. European Archives of Psychiatry and Clinical Neuroscience. 2023;273(3):699–717.<https://doi.org/doi:10.1007/s00406-022-01446-0>

93.	Richardson G, Day N, Goldschmidt L. Prenatal alcohol, marijuana, and tobacco use: infant mental and motor development. Neurotoxicology and teratology. 1995;17(4):479–87.<https://doi.org/doi:10.1016/0892-0362(95)00006-d>

94.	Richardson GA, Ryan C, Willford J, Day NL, Goldschmidt L. Prenatal alcohol and marijuana exposure: Effects on neuropsychological outcomes at 10 years. Neurotoxicology and Teratology. 2002;24(3):311–20.<https://doi.org/doi:10.1016/S0892-0362(02)00193-9>

95.	Roffman J, Sipahi E, Dowling K, Hughes D, Hopkinson C, Lee H, et al. Association of adverse prenatal exposure burden with child psychopathology in the Adolescent Brain Cognitive Development (ABCD) Study. PloS one. 2021;16(4):e0250235.<https://doi.org/doi:10.1371/journal.pone.0250235>

96.	Rose-Jacobs R, Soenksen S, Appugliese D, Cabral H, Richardson M, Beeghly M, et al. Early adolescent executive functioning, intrauterine exposures and own drug use. Neurotoxicology and teratology. 2011;33(3):379–92.<https://doi.org/doi:10.1016/j.ntt.2011.02.013>

97.	Rose-Jacobs R, Augustyn M, Beeghly M, Martin B, Cabral HJ, Heeren TC, et al. Intrauterine substance exposures and Wechsler Individual Achievement Test-II scores at 11 years of age. Vulnerable Children and Youth Studies. 2012;7(2):186–97.<https://doi.org/doi:10.1080/17450128.2011.648967>

98.	Rose-Jacobs R, Richardson M, Buchanan-Howland K, Chen C, Cabral H, Heeren T, et al. Intrauterine exposure to tobacco and executive functioning in high school. Drug and alcohol dependence. 2017;176:169–75.<https://doi.org/doi:10.1016/j.drugalcdep.2017.02.022>

99.	Ryan JE, McCabe SE, Wilens TE, Weigard A, Worster B, Veliz P. Prenatal polysubstance use and attention-deficit/hyperactivity disorder (ADHD). American Journal of Preventive Medicine. 2024;66(3):548–50.<https://doi.org/doi:10.1016/j.amepre.2023.11.023>

100.	Salzwedel A, Chen G, Chen Y, Grewen K, Gao W. Functional dissection of prenatal drug effects on baby brain and behavioral development. Human brain mapping. 2020;41(17):4789–803.<https://doi.org/doi:10.1002/hbm.25158>

101.	Serino MD, Peterson MB, Rosen MT. Psychological Functioning of Women Taking Illicit Drugs during Pregnancy and the Growth and Development of Their Offspring in Early Childhood. Journal of dual diagnosis. 2018;14(3):158–70.<https://doi.org/doi:10.1080/15504263.2018.1468946>

102.	Singer L, Arendt R, Farkas K, Minnes S, Huang J, Yamashita T. Relationship of prenatal cocaine exposure and maternal postpartum psychological distress to child developmental outcome. Development and psychopathology. 1997;9(3):473–89.<https://doi.org/doi:10.1017/s0954579497001259>

103.	Smid M, Metz T, McMillin G, Mele L, Casey B, Reddy U, et al. Prenatal Nicotine or Cannabis Exposure and Offspring Neurobehavioral Outcomes. Obstetrics and gynecology. 2022;139(1):21–30.<https://doi.org/doi:10.1097/AOG.0000000000004632>

104.	Tadesse A, Ayano G, Dachew B, Betts K, Alati R. Exposure to maternal cannabis use disorder and risk of autism spectrum disorder in offspring: A data linkage cohort study. Psychiatry research. 2024;337:115971.<https://doi.org/doi:10.1016/j.psychres.2024.115971>

105.	Tadesse A, Dachew B, Ayano G, Betts K, Alati R. Maternal cannabis use disorder and offspring behavioral outcomes: findings from a linked data cohort study. Psychiatry research. 2025;346:116404.<https://doi.org/doi:10.1016/j.psychres.2025.116404>

106.	Talavera-Barber M, Morehead E, Ziegler K, Hockett C, Elliott A. Prenatal cannabinoid exposure and early language development. Frontiers in pediatrics. 2023;11:1290707.<https://doi.org/doi:10.3389/fped.2023.1290707>

107.	Tchuente V, Sheehy O, Zhao J, Gorgui J, Gomez Y, Berard A. Is in-utero exposure to cannabis associated with the risk of attention deficit with or without hyperactivity disorder? A cohort study within the Quebec Pregnancy Cohort. BMJ open. 2022;12(8):e052220.<https://doi.org/doi:10.1136/bmjopen-2021-052220>

108.	Tennes K, Avitable N, Blackard C, Boyles C, Hassoun B, Holmes L, et al. Marijuana: prenatal and postnatal exposure in the human. NIDA research monograph. 1985;59:48–60.<https://doi.org/doi>:

109.	Vishnubhotla R, Ahmad S, Zhao Y, Radhakrishnan R. Impact of prenatal marijuana exposure on adolescent brain structural and functional connectivity and behavioural outcomes. BRAIN COMMUNICATIONS. 2024;6(2).<https://doi.org/doi:10.1093/braincomms/fcae001>

110.	Watts D, Lebel C, Chaput K, Giesbrecht G, Dewsnap K, Baglot S, et al. Evaluation of the Association Between Prenatal Cannabis Use and Risk of Developmental Delay. JAACAP OPEN. 2024;2(4):250–62.<https://doi.org/doi:10.1016/j.jaacop.2024.03.004>

111.	Winiger E, Hewitt J. Prenatal cannabis exposure and sleep outcomes in children 9-10 years of age in the adolescent brain cognitive development (SM) study. Sleep health. 2020;6(6):787–9.<https://doi.org/doi:10.1016/j.sleh.2020.05.006>

112.	Young-Wolff K, Kong K, Alexeeff S, Croen L, Oberman N, Kirane H, et al. Prenatal Cannabis Use and Offspring Attention Deficit Hyperactivity Disorder and Disruptive Behavior Disorders: A Retrospective Cohort Study. Journal of developmental and behavioral pediatrics : JDBP. 2025;46(1):e25–e32.<https://doi.org/doi:10.1097/DBP.0000000000001323>
